# Expanded LUXendin Color Palette for GLP1R Detection and Visualization In Vitro and In Vivo

**DOI:** 10.1021/jacsau.2c00130

**Published:** 2022-04-04

**Authors:** Julia Ast, Alissa N. Novak, Tom Podewin, Nicholas H. F. Fine, Ben Jones, Alejandra Tomas, Ramona Birke, Kilian Roßmann, Bettina Mathes, Jenny Eichhorst, Martin Lehmann, Amelia K. Linnemann, David J. Hodson, Johannes Broichhagen

**Affiliations:** † Institute of Metabolism and Systems Research (IMSR), and Centre of Membrane Proteins and Receptors (COMPARE), University of Birmingham, Birmingham B15 2TT, U.K.; ‡ Centre for Endocrinology, Diabetes and Metabolism, Birmingham Health Partners, Birmingham B15 2TT, U.K.; § Department of Pediatrics, and Indiana Center for Diabetes and Metabolic Diseases, Indiana University School of Medicine, Indianapolis, Indiana 46202, United States; ∥ Department of Chemical Biology, Max Planck Institute for Medical Research, Heidelberg 69120, Germany; ⊥ Section of Endocrinology and Investigative Medicine, Division of Diabetes, Endocrinology and Metabolism, Imperial College London, London W12 0NN, U.K.; # Section of Cell Biology and Functional Genomics, Division of Diabetes, Endocrinology and Metabolism, Imperial College London, London W12 0NN, U.K.; ¶ Leibniz-Forschungsinstitut für Molekulare Pharmakologie, Berlin 13125, Germany; ∇ Department of Pharmacology and Cell Biology, Leibniz-Forschungsinstitut für Molekulare Pharmakologie, Berlin 13125, Germany; ○ Oxford Centre for Diabetes, Endocrinology and Metabolism (OCDEM), NIHR Oxford Biomedical Research Centre, Churchill Hospital, Radcliffe Department of Medicine, University of Oxford, Oxford OX3 7LE, U.K.

**Keywords:** incretin, GLP1R, diabetes, beta cell, fluorescent probes, noninvasive imaging

## Abstract

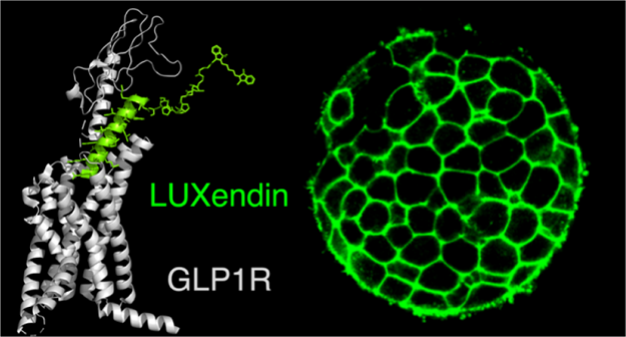

The glucagon-like peptide-1 receptor (GLP1R) is expressed in peripheral tissues and the brain, where it exerts pleiotropic actions on metabolic and inflammatory processes. Detection and visualization of GLP1R remains challenging, partly due to a lack of validated reagents. Previously, we generated **LUXendins**, antagonistic red and far-red fluorescent probes for specific labeling of GLP1R in live and fixed cells/tissues. We now extend this concept to the green and near-infrared color ranges by synthesizing and testing **LUXendin492**, **LUXendin551**, **LUXendin615**, and **LUXendin762**. All four probes brightly and specifically label GLP1R in cells and pancreatic islets. Further, **LUXendin551** acts as a chemical beta cell reporter in preclinical rodent models, while **LUXendin762** allows noninvasive imaging, highlighting differentially accessible GLP1R populations. We thus expand the color palette of **LUXendins** to seven different spectra, opening up a range of experiments using wide-field microscopy available in most labs through super-resolution imaging and whole animal imaging. With this, we expect that **LUXendins** will continue to generate novel and specific insights into GLP1R biology.

## Introduction

The glucagon-like peptide-1 receptor (GLP1R) is a class B G protein-coupled receptor involved in the regulation of glucose homeostasis, food intake, and inflammation.^1,2^ As such, GLP1R agonist (GLP1RA) therapy has become a mainstay of type-2 diabetes treatment during the past decade, with a number of drugs on the market based upon stabilized analogues of glucagon-like peptide-1.^3^ Most recently, phase III trials of the third-generation semaglutide have shown a ∼15% reduction in body weight when combined with lifestyle interventions,^4^ leading to the approval of GLP1RAs as the first nonsurgical treatment for complex obesity. Despite this, information concerning the localization of GLP1R is lacking, primarily due to the paucity of reliable and specific reagents for its detection and visualization.^5^ Without this knowledge, it is difficult to elucidate the exact cellular mechanisms underlying GLP1R actions, many of which could be key to developing even more specific or effective treatments for metabolic/inflammatory disease states, for instance, by tissue-targeted delivery.^6^ For example, GLP1RAs have been shown to reduce the progression from nonalcoholic fatty liver disease/nonalcoholic steatohepatitis to fulminant fibrosis,^7,8^ yet where and how the GLP1R acts is currently uncertain. Along similar lines, GLP1RAs exert inhibitory (and beneficial) effects on glucagon secretion, yet pancreatic GLP1R distribution and signaling remain debated.^5^ Lastly, the neural circuits that GLP1RAs are able to access to exert effects on food intake remain to be fully delineated.^9−11^

Reagents to detect GLP1R in tissues include antibodies, reporter mice, and fluorescent ligands.^5^ Historically, studies with antibodies have been confounded by the use of nonspecific antisera, which detect non-GLP1R targets.^12,13^ Four specific antibodies now exist and have been extensively validated, including in the GLP1R knockout tissue, or cells heterologously expressing human GLP1R.^14^ However, the available antibodies do not perform well for immunofluorescence staining in the brain and cannot be used for live visualization of the GLP1R using microscopy. Reporter mice, where cells that express(ed) GLP1R are selectively labeled with high fidelity, have been used to address this limitation, demonstrating excellent concurrence with other approaches.^15,16^ However, reporter alleles neither visualize the receptor itself nor differentiate cells that once expressed GLP1R, but no longer do so (the cell will be indelibly marked). Fluorescent agonists bind the GLP1R orthosteric site in live tissues and can also be fixed to allow further immunohistochemical analysis.^10,17,18^ However, this approach is confounded by activation of GLP1R, and as such the unstimulated fraction cannot be studied in live cells.

Recently, we have developed fluorescent antagonists, which are capable of detecting GLP1R in its unstimulated/antagonized state in the membrane.^19^ Advantageously, these probes, termed **LUXendins**, are equipotent to native antagonists, work well in the periphery and brain, display excellent brightness, and can be formalin-fixed.^19^ To date, **LUXendins** have been freely and widely distributed to dozens of other labs for academic use,^20−22^ opening up new GLP1R biology. The **LUXendins** were necessarily furnished with red and far-red fluorophores, not only allowing conventional microscopy but also for the aims of our study, total internal reflection (TIRF) microscopy and stimulated emission depletion (STED) nanoscopy.^19^ Aiming for more experimental modalities and taking on board comments from end users, we now expand the color palette of the **LUXendins**, further increasing their utility for wide-field, confocal, intravital, and near-infrared microscopy, allowing imaging from the single cell to the whole animal.

## Results

### Design and Synthesis of **LUXendin492**, **LUXendin551**, **LUXendin615**, and **LUXendin762**

**Figure 1:**
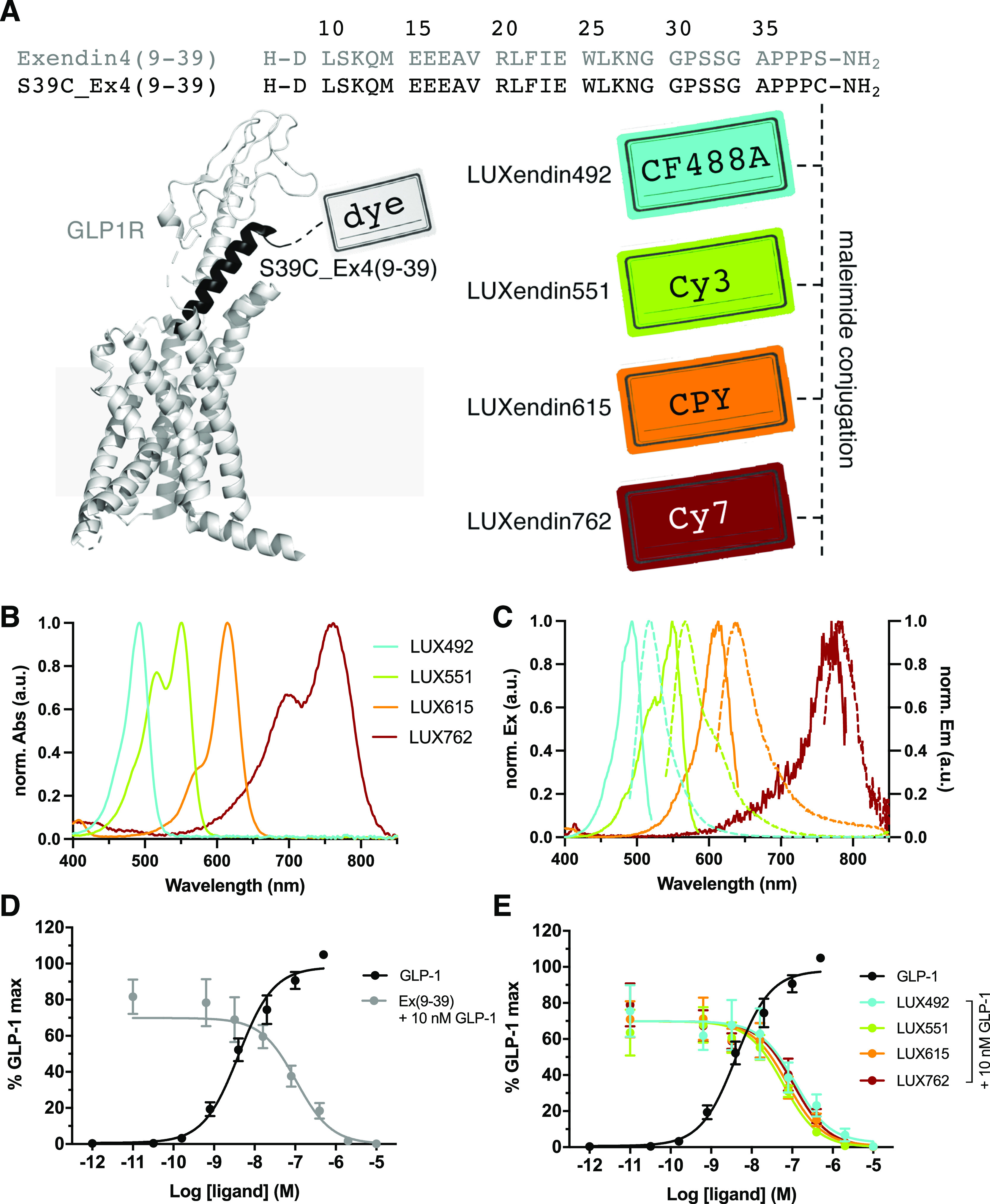
Sequence, structure, photophysical properties, and pharmacology of **LUXendin492**, **LUXendin551**, **LUXendin615**, and **LUXendin762**. (A) **LUXendins** are based on the antagonist Exendin4(9–39) with a S39C mutation to install fluorophores *via* late-stage thiol–maleimide chemistry. The model shows GLP1R in complex with a peptide ligand [pdb: 5VAI, cartoon obtained by the in-built building capability of PyMOL (Palo Alto, CA, USA)]. CF488A, Cy3, CPY, and Cy7 were installed as fluorescent labels to give **LUXendin492**, **LUXendin551**, **LUXendin615**, and **LUXendin762**, respectively. (B) UV/vis spectra of novel **LUXendins**. (C) Fluorescence excitation and emission spectra of **LUXendins**. (D) cAMP response in GLP1R-transfected HEK293 cells for GLP-1 (agonist, black) and Ex(9–39) (antagonist) in the presence of 10 nM GLP-1 (gray) (*n* = 6 independent repeats). (E) Sames as (D), but in response to **LUXendins** (colored), showing the antagonistic nature of the probes.

Exendin4(9–39) was employed as a scaffold for modification with fluorophores. Using solid-phase peptide synthesis (SPSS), exendin4(9–39)-S39C (S39C-Ex4) was generated, bearing a C-terminal serine to cysteine substitution for functionalization *via* the introduced thiol handle. CF488A-, Cy3-, CPY-, and Cy7-conjugated versions were produced using cysteine–maleimide reactions and termed **LUXendin492**, **LUXendin551**, **LUXendin615**, and **LUXendin762**, respectively (Figure 1A), according to their maximal absorption values. Spectral properties were determined using UV/vis and fluorescence spectroscopy (Figure 1B,C) (Table 1) and were in line with known properties of the fluorophores used, for which extinction coefficients and quantum yields are reported. Full compound characterization and purity assessment are provided in the Supporting Information.

**Table 1:** Spectral Properties of GLP1R Labeling Probes^a^

	dye	λEx/nm	λEm/nm	ε^b^/M–^1^ cm–^1^	Φ
**LUXendin492**	CF488A	492	517	70,000^d^	N/A
**LUXendin551**	Cy3	551	567	150,000^e^	0.31
**LUXendin555**^c^	TMR	555	579	84,000	0.31
**LUXendin615**	CPY	615	640	100,000^23^	0.59
**LUXendin645**^c^	Cy5	645	664	250,000	0.22
**LUXendin651**^c^	SiR	651	669	100,000	0.43
**LUXendin762**	Cy7	762	784	199,000^e^	0.30

a Maximal excitation and emission wavelengths, extinction coefficients, and quantum yields of all fluorophores used for making the **LUXendin** probes.

b For maleimide-conjugated fluorophores.

c Previous study.

d https://biotium.com/technology/cf-dyes/cf488a-dye/.

e https://de.lumiprobe.com.

### **LUXendin492**, **LUXendin551**, **LUXendin615**, and **LUXendin762** Are Potent GLP1R Antagonists

We first assessed the antagonist activity of the novel **LUXendins** using cAMP assays in SNAP-GLP1R:HEK293 cells. As expected, native GLP1(7–36)NH_2_ increased intracellular cAMP levels with a pEC_50_ = 8.3 ± 0.2 (Figure 1D). Application of increasing doses of the benchmark antagonist Exendin4(9–39) inhibited GLP1-stimulated cAMP levels with a pIC_50_ = 7.0 ± 0.2 (Figure 1D). Confirming that the installed fluorophores did not alter potency of the Exendin4(9–39)-S39C backbone, **LUXendin492** (pIC_50_ = 7.2 ± 0.2), **LUXendin551** (pIC_50_ = 7.2 ± 0.1), **LUXendin615** (pIC_50_ = 7.2 ± 0.1), and **LUXendin762** (pIC_50_ = 7.0 ± 0.2) all inhibited GLP1-stimulated (10 nM) cAMP levels in a manner equipotent to Exendin4(9–39) (Figure 1E). The pharmacology of Exendin4(9–39)-S39C has previously been determined.^19^ Thus, the novel **LUXendins** show indistinguishable antagonistic properties from Exendin4(9–39) in terms of cAMP signaling. With this in mind, we set out to study novel **LUXendin** labeling in cells and tissues, as well as the whole organism.

### **LUXendin492**, **LUXendin551**, and **LUXendin615** Specifically Label GLP1R

**Figure 2:**
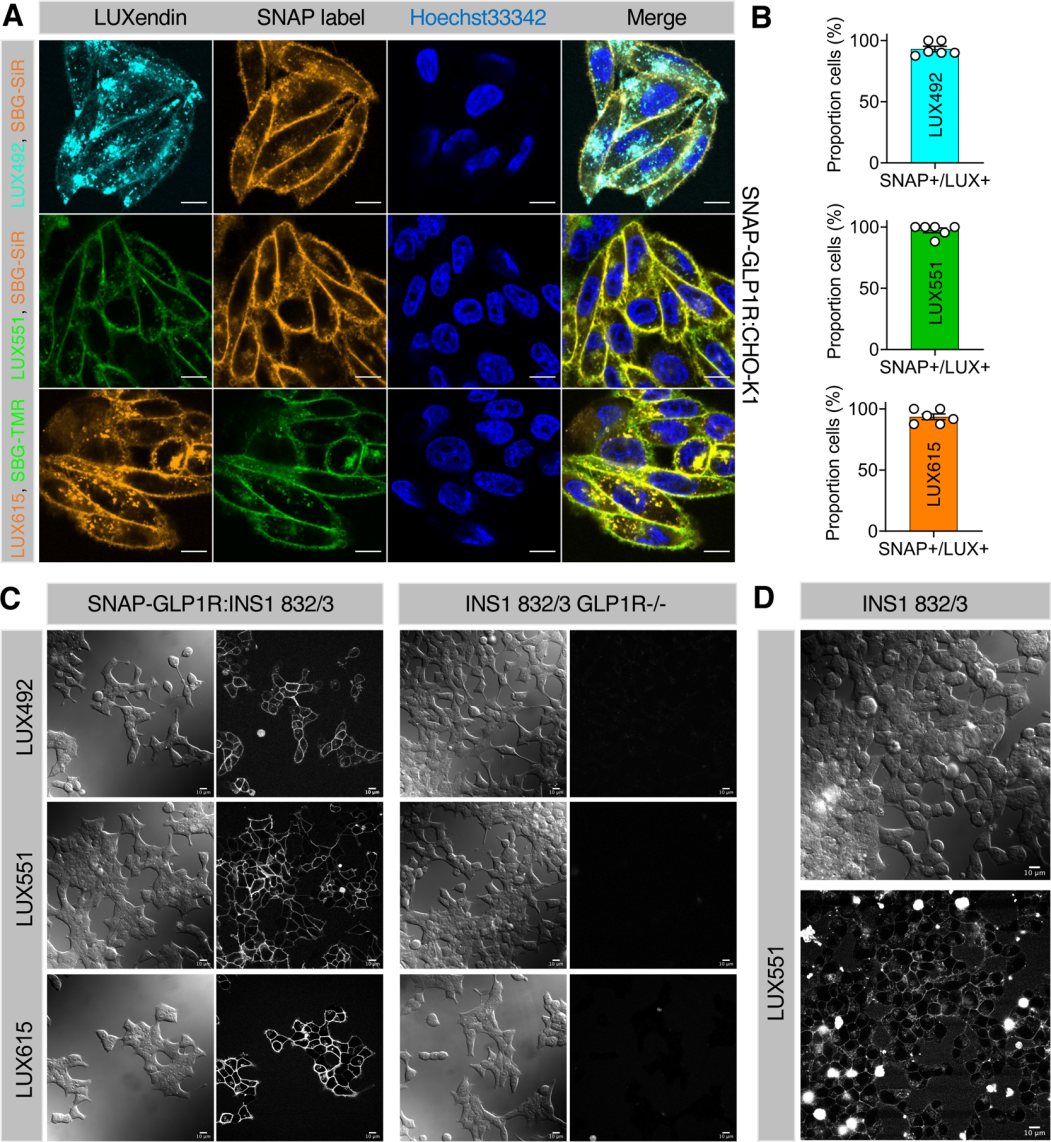
(A) Labeling of live cells with **LUXendin492**, **LUXendin551**, and **LUXendin615**. SNAP-GLP1R:CHO-K1 cells were incubated with **LUXendin492** (LUX492), **LUXendin551** (LUX551), and **LUXendin615** (LUX615) before orthogonal SNAP-labeling with either cell-impermeable SBG-TMR or SBG-SiR and confocal imaging (nuclei were stained using Hoechst33342) (scale bar = 10 μm) (*n* = three images from experiments performed in duplicate). (B) Labeling efficiency of **LUXendin492**, **LUXendin551**, and **LUXendin615** in SNAP-GLP1R:CHO-K1 cells, quantified as the proportion of SNAP^+^/LUX^+^ cells (versus total SNAP-labeled cells) (*n* = 6 wells). (C) **LUXendin492**, **LUXendin551**, and **LUXendin615** label SNAP-GLP1R:INS1 832/3 but not INS1 832/3 GLP1R^–/–^ cells. (D) Additionally, endogenous GLP1R can be visualized with **LUXendin551** in INS1 832/3 cells, which express the receptor at lower levels than in islets (scale bars = 10 μm).

To establish the labeling efficacy and specificity of the novel **LUXendins**, SNAP-GLP1R:CHO-K1 cells were incubated with each probe, before washing and orthogonal SNAP labeling with cell impermeable SBG-TMR or SBG-SiR.^24^ High-resolution confocal images showed predominantly membrane-localized **LUXendin** staining in SNAP-GLP1R:CHO-K1 cells, which overlapped with labeling of the SNAP-tag located on the GLP1R N-terminus (Figure 2A). Labeling efficiency was close to 100% for all probes investigated (Figure 2B). No signal was detected in mock (nontransfected) CHO-K1 cell controls (Supporting Information, Figure S1). **LUXendins** were also able to label stably transfected SNAP-GLP1R:INS1 832/3 rat beta cells (Figure 2C), as well as native INS1 832/3, which endogenously express GLP1R (Figure 2D). Demonstrating high specificity, the signal was absent in INS1 832/3 GL1PR^–/–^ cells, CRISPR deleted for the GL1PR (Figure 2C). Of note, **LUXendin492** and **LUXendin615** staining was less “clean” than **LUXendin551**, with some fluorescent signals present in the cytoplasm. We have previously reported a similar staining distribution for **LUXendin555** (TMR) *versus***LUXendin645** (Cy5),^19^ demonstrating a general preference toward cyanine-based dyes over their xanthene-based counterparts for cell labeling. To gain further insight into this observation, we applied **LUXendin492** and **LUXendin615** to SNAP-GLP1R:CHO-K1 cells, in parallel with cell-permeable SNAP labels^24^ (Supporting Information, Figure S2). A similar experiment was performed but using cell impermeable SNAP labels,^25^ before chasing with **LUXendin492** and **LUXendin615** (Supporting Information, Figure S3). In both cases, no overlap with SNAP label was noticed, suggesting that intracellular **LUXendin492** and **LUXendin615** staining patterns are unlikely to stem from bound GLP1R. Nonetheless, all the **LUXendins** tested clearly label membrane **GLP1R**.

We next validated **LUXendins** for use in wide-field microscopy, which is widely available in most labs, serves to illustrate the robustness of labeling, and has the added advantage of allowing detection of near-infrared probes using cost efficient and fast switchable LED excitation and sensitive sCMOS detectors. As for confocal imaging, a similar pattern of **LUXendin492**, **LUXendin551**, and **LUXendin615** staining was seen, with the cyanine-based dye (LUX551) performing superiorly (Supporting Information, Figure S4).

### **LUXendin492**, **LUXendin551**, and **LUXendin615** Specifically Label Endogenous GLP1R

**Figure 3:**
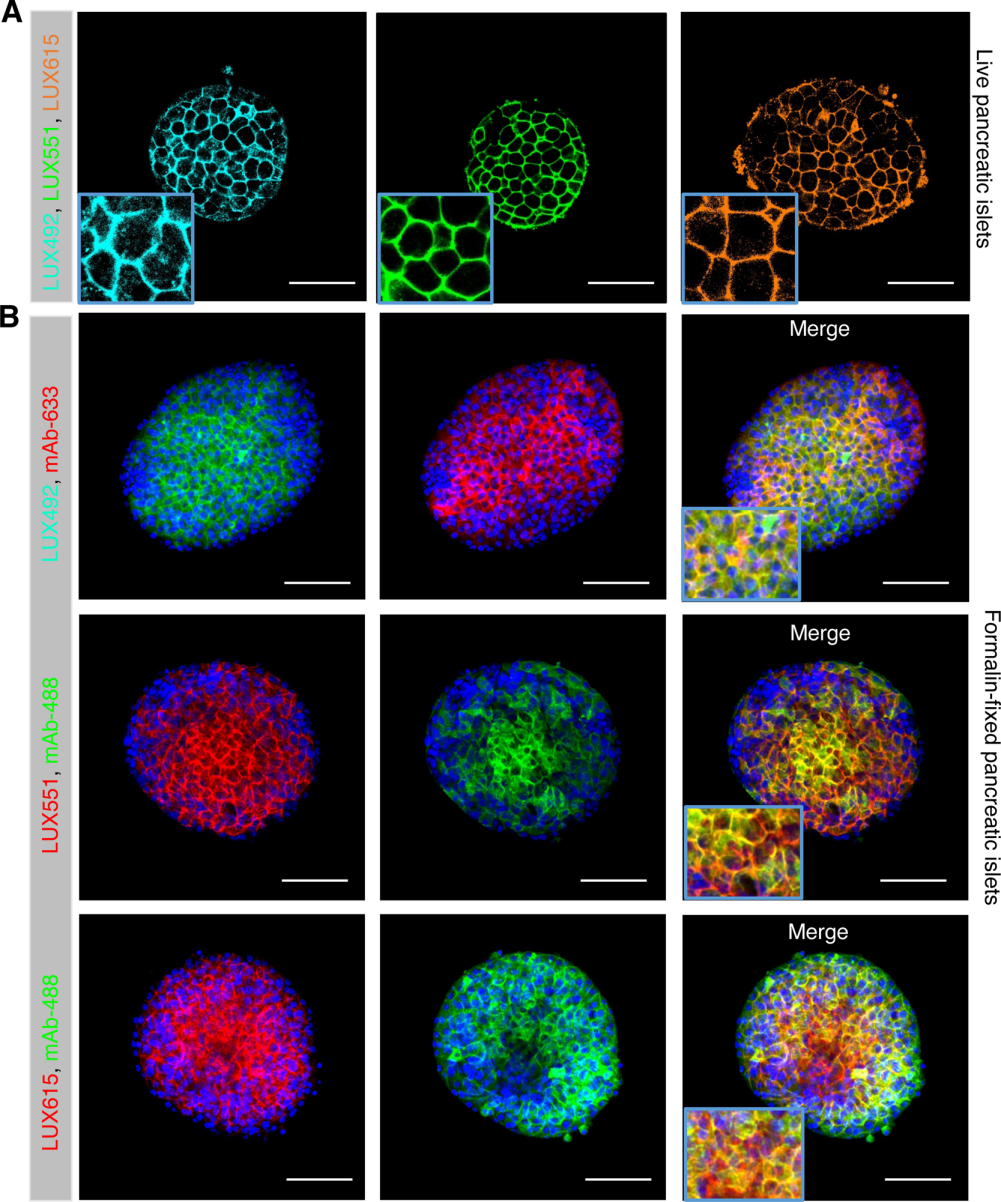
Labeling of live and fixed islets of Langerhans with **LUXendin492**, **LUXendin551**, and **LUXendin615**. (A) Incubation of live islets with **LUXendin492** (LUX492), **LUXendin551** (LUX551), or **LUXendin615** (LUX615) leads to bright staining confined to the cell membrane (scale bar = 53 μm) (*n* = 11–13 islets from 4 mice). (B) **LUXendin492**, **LUXendin551**, and **LUXendin615** signals can still be detected following formalin fixation and are co-localized with orthogonal emission from a specific monoclonal antibody against GLP1R (mAb) (scale bar = 85 μm) (*n* = 9–10 islets from 4 mice).

One of the major advantages of **LUXendins** is that they can be used to visualize GLP1R in both live and fixed complex tissues. Pancreatic islets of Langerhans served as the testbed for the novel **LUXendins** because they express GLP1R, which is predominantly localized to the beta cell compartment.^5,19^ Following 1 h of incubation with **LUXendin492**, **LUXendin551**, and **LUXendin615**, intense labeling was observed throughout the islet, with large gaps apparent (presumably representing the GLP1R-negative alpha cell compartment, which comprises ∼20% of the rodent islet, as reported^19^) (Figure 3A). In all cases, labeling with the novel **LUXendins** could still be observed following formalin-fixation (Figure 3B), further expanding the utility of the novel **LUXendins** for protein identification together with immunohistochemistry. Confirming specificity, **LUXendin492**, **LUXendin551**, and **LUXendin615** signals co-localized with specific GLP1R monoclonal antibody staining (Novo Nordisk 7F38, fully validated in GLP1R^–/–^ tissue^19^) (Figure 3B).

### **LUXendin551** Allows In Vivo Fluorescent Labeling of Islets in NOD Mice

The NOD mouse is a type-1 diabetes model that develops insulitis at 4–8 weeks of age, with frank diabetes occurring from 30 weeks of age.^26^ However, identifying beta cells during disease trajectory is challenging because the polygenic NOD genetic background cannot be easily recombined with common inbred beta cell reporter strains (e.g., Ins1Cre; R26YFP). We and others have previously shown that GLP1R expression is beta cell specific^16,19^ and we thus hypothesized that **LUXendins** might open up the possibility to identify beta cells in NOD (and other polygenic) mice.

**Figure 4:**
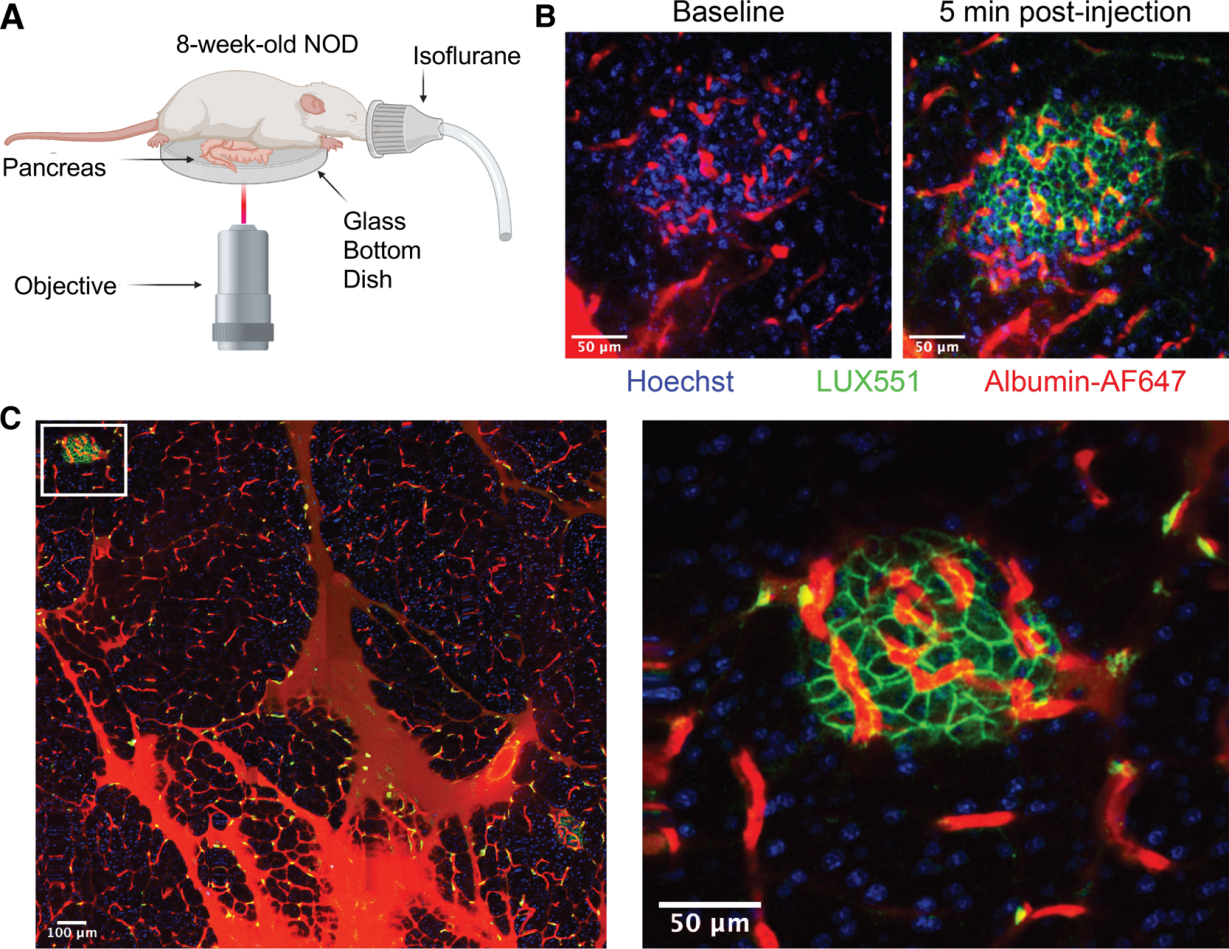
Labeling of GLP1R in NOD mouse islets *in vivo*. (A) Two-photon intravital imaging schematic for visualization of the exposed intact pancreas in an 8-week-old NOD mouse. (B) Representative image collected at baseline and 5 min post-injection, showing islet vasculature and accumulation of **LUXendin551** at cell membranes (scale bar = 50 μm). (C) Mosaic image of externalized pancreas (scale bar = 100 μm) and the enlarged islet within this region (scale bar = 50 μm).

To investigate this, the pancreas was exposed in 8-week-old anesthetized NOD mice through a small abdominal incision before being subjected to two-photon microscopy (Figure 4A). Baseline images were acquired following retro-orbital injection of Hoechst33342 and albumin-AF647 to label the nuclei and vasculature, respectively. Prior to **LUXendin551** injection there was no detectable signal (Figure 4B). Rapid labeling occurred following the administration of **LUXendin551** and was detected for at least 30 min post-injection (Figure 4B). These studies also demonstrated that **LUXendin551** is highly specific to islets and provides the ability to distinguish islets and beta cells from exocrine tissue (Figure 4C).

### **LUXendin762** Allows Noninvasive Fluorescence Detection of GLP1R In Vivo

**Figure 5:**
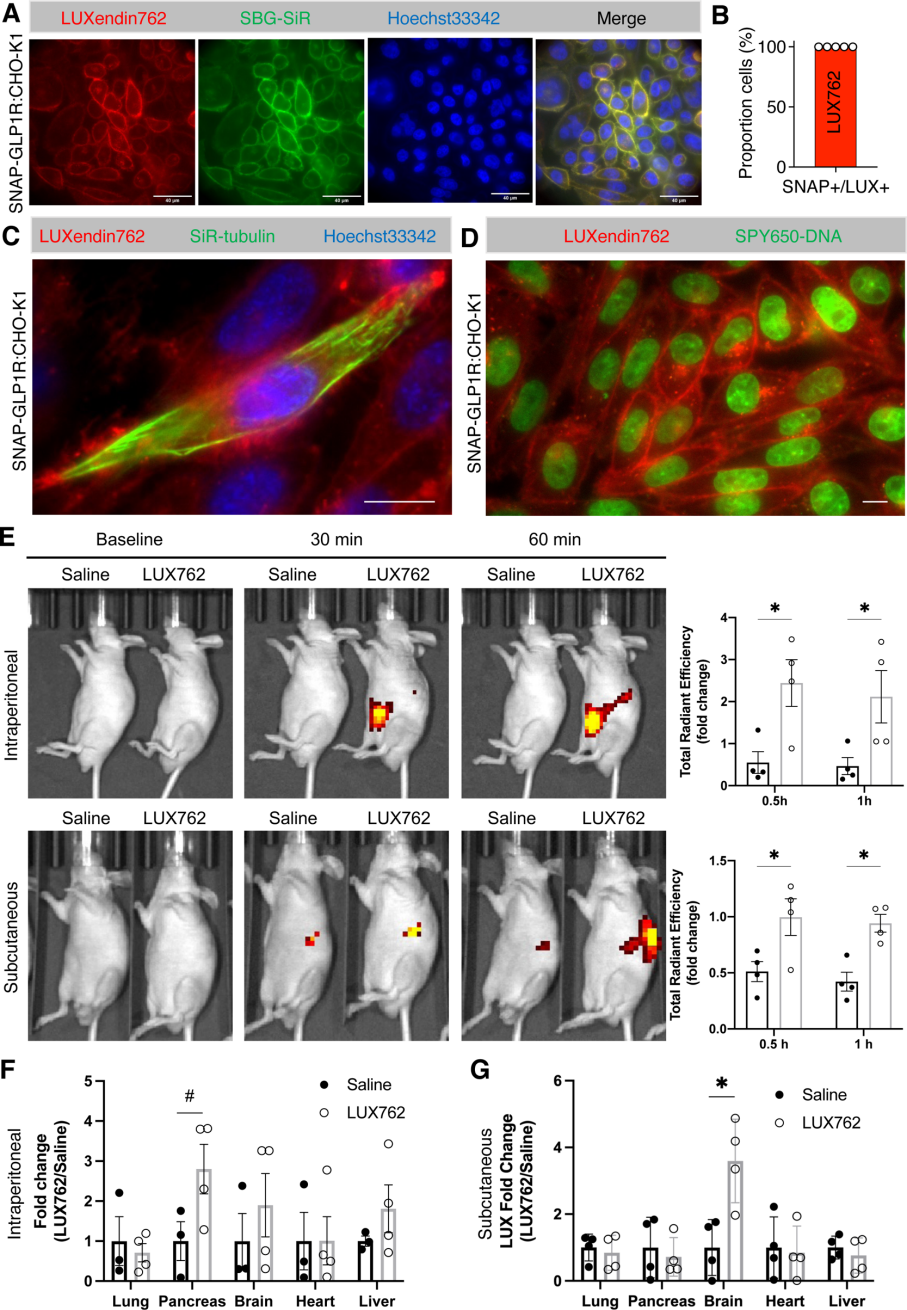
Evaluation of **LUXendin762** distribution *in vivo*. (A) **LUXendin762** (LUX762, 200 nM) labels the membrane of SNAP-GLP1R:CHO-K1 cells (nuclei were stained using Hoechst33342) (scale bar = 40 μm) (*n* = 3 independent experiments). (B) Labeling efficiency of **LUXendin762** in SNAP-GLP1R:CHO-K1 cells (*n* = 5 wells). (C) **LUXendin762** is compatible with far-red SiR-tubulin that labels microtubules of SNAP-GLP1R:CHO-K1 cells (nuclei were stained using Hoechst33342; please note that not all cells were stained by SiR-tubulin) (scale bar = 10 μm). (D) **LUXendin762** is compatible with far-red SPY650-DNA that labels nuclei of SNAP-GLP1R:CHO-K1 cells (scale bar = 10 μm). (E) *In vivo* images of mice intraperitoneally or subcutaneously injected with saline or **LUXendin762** at the baseline and 30 min and 1 h post-injection. Data plotted as the fold change of total radiant efficiency signals of the whole body measured at 30 min and 1 h post-injection. (F) *Ex vivo* analysis of harvested tissues 1 h post-intraperitoneal injection (*n* = 4 mice). (G) *Ex vivo* analysis of tissues 1 h post-subcutaneous injection (*n* = 4 mice). Graphs show mean ± SEM. #*p* = 0.08, *p < 0.05 (unpaired *t*-test for each tissue).

Due to its near-infrared excitation, we surmised that a Cy7-linked GLP1R antagonist, **LUXendin762**, might allow intravital labeling of GLP1R, using the widely available and noninvasive IVIS *in vivo* imaging systems. We first tested **LUXendin762***in cellulo* in SNAP-GLP1R:CHO-K1 cells and in keeping with its pharmacology were able to detect strong membrane labeling, with little evidence of intracellular accumulation, again pointing to the high performance of cyanine-based dyes (Figure 5A). Quantifying staining against SNAP-positive cells labeled with SBG-SiR, we found maximal efficiency (Figure 5B), that is, all cells were positive for both stains (also see Supporting Information, Figure S5). **LUXendin762** was next used to label primary islets, again showing cell membrane localization (Supporting Information, Figure S6A), shown to be GLP1R-positive using validated monoclonal antibodies (Supporting Information, Figure S6B). No spectral overlap could be detected between Cy5 (**LUXendin645**) and Cy7 (**LUXendin762**) channels (Supporting Information, Figure S6A,B). Freeing the far-red channel allowed us to perform multicolor experiments with commercially available far-red SiR-tubulin (Figure 5C) and SPY650-DNA probes (Figure 5D) that mark microtubule and DNA structures, respectively, providing further possibilities for cellular imaging.

Confident that **LUXendin762** was able to specifically label GLP1R, we next injected Nude mice with the probe before imaging. A strong fluorescent signal could be detected in the abdomen and brain at 30 and 60 min, following intraperitoneal or subcutaneous injection, with fluorescence levels ∼2–5-fold higher than in animals receiving saline vehicle (Figure 5E). Because the signal intensity from the injection site is far brighter than in the brain, various organs were harvested. The pancreas of mice receiving intraperitoneal **LUXendin762** showed the highest fluorescent signal, while the brain, lung, heart, and liver were similar to saline-treated controls (Figure 5F, Supporting Information, Figure S7). By contrast, mice receiving subcutaneous **LUXendin762** displayed the highest probe levels in the brain, whereas no signal was detected in the pancreas, lung, and heart *versus* saline-treated control (Figure 5G, Supporting Information, Figure S7). Notably, the brain and pancreas are known to be GLP1R-positive,^5^ whereas the GLP1R is only expressed in small cell populations (or absent) in the lung, kidney, liver, and heart (e.g., smooth muscle of arterioles).^27,28^ Together, these studies show that **LUXendin762** can be detected *in vivo* in the whole organism and reveal a novel role for the injection route in determining GLP1R access.

## Discussion

In the present study, we synthesize and validate **LUXendin492**, **LUXendin551**, **LUXendin615**, and **LUXendin762**, antagonist probes spanning green to near infrared for the visualization of GLP1R in cells, tissues, and animals. Together with our previous **LUXendin555**, **LUXendin645**, and **LUXendin651** probes,^19^ we now extend the **LUXendin** color palette to seven different spectra. These probes contain a range of different fluorophores suitable for wide-field, confocal, super-resolution, intravital microscopy, and small animal optical imaging, as well as FACS.

Pharmacologically, the novel **LUXendins** behave as full antagonists at the GLP1R, with similar potency to benchmark Exendin4(9–39). These studies further validate the robustness of the synthetic approach used and highlight the advantages of the S39C C-terminally substituted backbone used previously for Exendin4(9–39)^19^ and Exendin4(1–39).^29^ We envisage that in the future a similar backbone might be amenable to functionalization with biotin, complexed lanthanides, singlet oxygen generators or even nanoparticles, for example to allow nonfluorescent labeling for mass spectrometry, magnetic resonance imaging, or electron microscopy. With our observation that cyanine fluorophores behave more “cleanly” for microscopy, we are eager to find out how other molecular markers and tracers behave, and these endeavors are ongoing in our laboratories.

Of note, labeling with the novel **LUXendins** was co-localized with both SNAP-GLP1R and specific monoclonal antibody staining, as expected given the previous thorough validation of **LUXendin555**, **LUXendin645**, and **LUXendin651** stablemates.^19^ Moreover, no **LUXendin** signal could be detected in INS1 832/3 cells CRISPR-deleted for the GLP1R. These data also confirm that the Exendin4-S39C scaffold tolerates a most fluorophores without significant effects on labeling or pharmacology. While some punctate staining was seen with non-cyanine dyes, this does not reflect GLP1R activation, since: (1) all **LUXendins** were potent antagonists; (2) no co-localization from intracellular signals were seen in SNAP-GLP1R cell systems; and (3) we showed that punctate **LUXendin** signal was not co-localized with GLP1R monoclonal antibody.^19^ By performing pulse-chase experiments using permeable and impermeable labels against SNAP-GLP1R, we further confirmed that punctate staining for **LUXendin492** and **LUXendin615** does not reflect activated GLP1R. One explanation for this observation could be preferred cellular uptake of xanthene-based **LUXendin492** and **LUXendin615** by macropinocytosis, a pathway for cells to uptake extracellular material caused by membrane ruffles. The presence of GLP1R is likely needed to increase local concentration of **LUXendin492**/**LUXendin615** at the cell surface because we did not see dye uptake in cells without GLP1R (mock-transfected). Indeed, recent studies have shown increased uptake of rhodamines when conjugated to peptidic, alpha-helical backbones.^30^ This is further supported by studies on fluorophore-labeled cell-penetrating peptides, in which rhodamines were found to exhibit a high hydrophobicity, leading to increased membrane penetration depth in liposomes.^31^ As such, we observed pronounced increases in performance of cyanine dyes (Cy3, Cy5, and Cy7) when compared to CF488, TMR, and CPY, most probably due to their molecular nature.

Using novel **LUXendins**, we were able to perform unprecedented experiments and reveal new biology regarding GLP1R. As the best performing dye, **LUXendin551** allowed GLP1R and thus beta cells to be reported in intravital experiments of a type-1 diabetes preclinical mouse model, which is not readily amenable to further genetic manipulation. Such experiments are important because we are still lacking information on the changes that occur in beta cell mass (and GLP1R expression) during insulitis and autoimmune destruction.^32^ To allow noninvasive imaging, Cy7 was installed on the **LUXendin** backbone to produce **LUXendin762**, a near-infrared probe. We were able to demonstrate that the **LUXendin762** signal can be recorded *in vivo* (compared to saline-treated controls) and sequesters in organs known to express the GLP1R such as the pancreas and brain.^5^ Of interest, **LUXendin762** highlighted differential access routes to peripheral and brain GLP1R sites, with subcutaneous and not intraperitoneal injection labeling the latter. While the mechanisms are currently unknown, we speculate that ligand injected subcutaneously is less prone to the first pass effect and as such is able to abundantly enter the carotid arteries for entry into the brain. **LUXendin762** thus opens up for the first time noninvasive longitudinal studies of GLP1R in mice using readily accessible platforms available in most academic/industrial animal facilities. In addition, increasing the **LUXendin762** dose, covering the injection sites, or using a more direct injection route (e.g., intracerebroventricular injection) might allow imaging of probe arrival in the pancreas and uptake in the brain. Such studies are particularly pertinent because GLP1R is also a readout for beta cell mass in preclinical models of type-2 diabetes and other metabolic syndromes.^33^ Furthermore, longitudinal measures in the same animal are statistically more powerful and refined compared to assessment of various timepoints in multiple cohorts.

In summary, a total of seven **LUXendins** now allow detection and labeling of GLP1R in five different colors, with fluorophores tailored for various imaging modalities. We anticipate that these specific and validated probes will provide further insights into GLP1R biology in the periphery and brain, with implications for treatment with GLP1RAs.

## Materials and Methods

### Synthesis

Exendin4(9–39)-S39C was generated as previously reported using solid phase peptide synthesis.^19,29^ TSTU activation of CPY-6-COOH and reaction with 1-(2-amino-ethyl)-pyrrole-2,5-dione (TFA salt, Aldrich) yielded Mal-CPY. Maleimide-conjugated CF488A (Aldrich), Cy3, and Cy7 (both Lumiprobe) were purchased from commercial vendors. Coupling to peptides was performed using thiol-maleimide chemistry in PBS, before characterization of novel compounds using HRMS, and purity (>95%) measurement using HPLC. Because fluorophores may exhibit environmental dependence upon receptor binding, for which extinction coefficients and quantum yields are challenging to determine, we instead highlight manufacturer measures for CF488-Mal, Cy3-Mal, CPY-6-COOH, and Cy7-Mal. In any case, all probes performed similarly when bound to SNAP-GLP1R or endogenous receptor, both in cells and tissues. Details for synthesis including characterization of **LUXendin492**, **LUXendin551**, **LUXendin615**, and **LUXendin762** are provided in the Supporting Information.

### Cell Culture

CHO-K1 cells stably expressing the human SNAP-GLP1R (Cisbio) (SNAP-GLP1R:CHO-K1) were maintained at 5% CO_2_, 37 °C in high-glucose phenol red Glutamax containing DMEM (Invitrogen, 31966047) supplemented with 10% heat-inactivated FCS (Invitrogen), 1% penicillin/streptomycin (Invitrogen), 500 μg/mL G418 (Invitrogen), 25 mM HEPES (Invitrogen), and 1% nonessential amino acids (Invitrogen), or DMEM (D6546, Sigma) supplemented with 10% FBS (Merck), 1% penicillin/streptomycin (Fisher Scientific), 500 μg/mL G418 (Fisher Scientific), 25 mM HEPES (Merck), 1% nonessential amino acids (Merck), and 2% l-glutamine (Thermo Scientific). The same medium without G418 was used to culture CHO-K1 cells. SNAP-GLP1R:HEK293 cells were cultured in DMEM supplemented with 10% FBS, 1% penicillin/streptomycin and 1 mg/mL G418. INS1 832/3 wild-type and GLP1R^–/–^ cells^34^ were cultured in RPMI supplemented with 11 mM glucose, 10% FCS, 10 mM HEPES, 2 mM l-glutamine, 1 mM pyruvate, 50 μM β-mercaptoethanol, and 1% penicillin/streptomycin and maintained as above. SNAP-GLP1R:INS1 832/3 cells were cultured as INS1 832/3 wild-type with the addition of 500 μg/mL G418.

### Animals

All studies with harvested tissue used 7–10 week old male C57BL6/J mice and were regulated by the Animals (Scientific Procedures) Act 1986 of the U.K (Personal Project Licenses P2ABC3A83 and PP1778740). Approval was granted by the University of Birmingham’s Animal Welfare and Ethical Review Body. All *in vivo* imaging experiments were performed with approval and oversight from the Indiana University Institutional Animal Care and Use Committee (IACUC).

### Islet Isolation

Animals were humanely euthanized using cervical dislocation, before injection of collagenase 1 mg/mL (Serva NB8) into the bile duct. Inflated pancreases were digested for 12 min at 37 °C and islets separated using a Ficoll (Sigma-Aldrich) gradient. Islets were cultured in RPMI medium containing 10% FCS, 100 units/mL penicillin, and 100 μg/mL streptomycin.

### cAMP Assays

cAMP assays were performed in SNAP-GLP1R:HEK293 cells, as previously described.^29^ Briefly, cells were incubated with 10 nM GLP-1(7–36)NH_2_ alongside increasing concentrations of **LUXendin** or Ex(9–39) for 30 min, before lysis and measurement of cAMP using a HTRF (Cisbio) assay, according to the manufacturer’s instructions. All assays were performed in the presence of 100–500 μM IBMX to inhibit phosphodiesterase activity. pEC_50_ and pIC_50_ values were calculated using log concentration–response curves fitted with a three- or four-parameter equation.

### Live Imaging

CHO-K1 and SNAP-GLP1R:CHO-K1 cells were seeded (60,000 cells/well) on microslide 8-well glass bottom dishes (ibidi, 80826) and grown for 2 days at 37 °C in a humidified 5% CO_2_ incubator. For imaging, cells were incubated for 30 min at 37 °C in a humidified 5% CO_2_ incubator in culture medium supplemented with 200 nM **LUXendin** and 5 μM Hoechst33342. SiR-tubulin (Spirochrome, SC002) and SPY650-DNA (Spirochrome, SC501) were used according to the manufacturer’s instructions with the exception that both probes were applied at 100-fold dilution. Cells were washed once in cell culture medium and imaged in live cell imaging buffer (Invitrogen, A14291DJ) at 37 °C and 5% CO_2_ using a Ti-E Nikon epifluorescence microscope equipped with pE4000 (cool LED), Penta Cube (AHF 66–615), 60× oil NA 1.49 (Apo TIRF Nikon), and imaged on a SCMOS camera (Prime 95B, Photometrics) operated by NIS Elements (Nikon). For excitation, the following wavelengths were used: **LUXendin492**: λ = 470 nm; **LUXendin551**: λ = 550 nm; **LUXendin615**: λ = 595 nm; **LUXendin645**: λ = 635 nm; and **LUXendin762**: λ = 740 nm.

For confocal imaging, CHO-K1 and SNAP-GLP1R:CHO-K1 were seeded in 96-well glass-bottom plates (Eppendorf, E0030741030) and kept at 37 °C and 5% CO_2_ until labeling in culture media supplemented with 200 nM **LUXendin** and 500 nM SNAP label at 37 °C, 5% CO_2_ for 30 min, and 4.4 μM Hoechst33342 for 5 min. For pulse-chase labeling, BG-Sulfo dyes were incubated for 30 min before the subsequent addition of **LUXendins** for another 30 min. After one wash, cells were imaged in culture media using an LSM880 meta-confocal microscope equipped with GaAsP spectral detectors and a 63× water NA 1.20 objective. For excitation/emission, the following wavelengths were used: Hoechst33324: λ = 405 nm/410–507 nm, **LUXendin492**: λ = 488 nm/490–560 nm, **LUXendin551** and SBG-TMR: λ = 561 nm/570–622 nm, and **LUXendin615** and SBG-SiR: λ = 633 nm/638–759 nm.

INS1 832/3, INS1 832/3 GLP1R^–/–^, and SNAP-GLP1R:INS1 832/3 cells were plated onto Mattek glass bottom dishes the day before imaging and imaged on a Zeiss LMS780 confocal microscope using a Plan-Apochromat 63× oil 1.40 NA objective for 2 min after addition of 100 nM LUXendin.

Islets were incubated with 100 nM **LUXendin492**, **LUXendin551**, or **LUXendin615** for 1 h at 37 °C in culture medium. Islets were washed three times and were imaged in culture medium using a Zeiss LSM880 AxioObserver microscope equipped with GaAsP spectral detectors and a 40× water NA 1.2 Korr FCS M27 objective. For excitation/emission, the following wavelengths were used: **LUXendin492**: λ = 488 nm/498–569 nm. **LUXendin551**: λ = 561 nm/569–667 nm. **LUXendin615**: λ = 633/641–694 nm.

### Immunostaining

Islets were incubated with 100 nM **LUXendin492**, **LUXendin551**, **LUXendin615**, and **LUXendin762** for 1 h at 37 °C in culture medium, before 4% formaldehyde fixation for 10 min. Mouse monoclonal anti-GLP1R 1:30 (Iowa DHSB; mAb #7F38) was applied overnight at 4 °C in PBS + 0.1% Triton + 1% BSA. Secondary antibodies were applied for 1 h at room temperature and included goat anti-mouse DyLight488 (excitation λ = 488 nm, emission λ = 489–552 nm) and goat anti-mouse Alexa Fluor 633 (excitation λ = 633 nm, emission λ = 641–694 nm). Samples were mounted on slides using Vectashield Hardset containing DAPI. Imaging was performed using a Zeiss LSM880 AxioObserver microscope, as above, for **LUXendin492**, **LUXendin551,** and **LUXendin615**, and using a Ti-E Nikon epifluorescence microscope, as above, for **LUXendin762**.

### Two-Photon *In Vivo* Imaging

Female NOD/ShiLtJ mice 8 weeks of age were anesthetized with isoflurane. A small, vertical incision was made to expose the intact pancreas. Then, the exposed pancreas was placed on a 50 mm glass-bottom dish for imaging on an inverted microscope. The body temperature was maintained using heating pads and heating elements on the objective. The mouse received, *via* retro-orbital injection, Hoechst 33342 (1 mg/kg in PBS) to label nuclei, albumin-AF647 (1 mg/kg in PBS) to label vasculature, and 75 μL of 30 μM **LUXendin551**. Images were collected using a Leica SP8 microscope, equipped with a 25×/0.95 NA objective and Spectra Physics MaiTai DeepSee multiphoton laser. Excitation was delivered at λ = 800 nm for Hoechst and Albumin-AF647, with signals collected at λ = 410–500 nm and λ = 550–590 nm, respectively. **LUXendin551** was excited at λ = 1050, with the signal collected at 650–700 nm. A conventional PMT was used for Hoechst, with a HyD detector used for Albumin-AF647 and **LUXendin551**. Blood was collected from the tail vein prior to and 30 min after **LUXendin555** injection, and glucose was measured using an AlphaTrak2 glucometer. After imaging, unconscious mice were euthanized by cervical dislocation.

### Noninvasive *In Vivo* Imaging

Whole body fluorescence accumulation and distribution was assessed in male athymic nude mice 8 weeks of age using an IVIS Spectral CT (Perkin Elmer). Mice were anesthetized with inhaled isoflurane and baseline images were acquired. Then, mice were intraperitoneally or subcutaneously injected with 100 μL of saline or 5 μM **LUXendin762**. Images were collected using a broad excitation and emission series combination ranging from 640 to 675 nm and 680 to 760 nm, respectively, at 30 min and 1 h post-injection. At the end point, animals were sacrificed, and tissues (pancreas, heart, brain, lung, and liver) were harvested for *ex vivo* fluorescence analysis. Spectral unmixing and quantification were analyzed using Living Image software.

## Supporting Information Available

Chemical synthesis, characterization, labeling of live CHO-K1 cells and fixed islets, wide-field imaging, and images of individual tissues (PDF)
