# Supplementary material for: Expanded LUXendin Color Palette for GLP1R Detection and Visualization In Vitro and In Vivo
Source: JACS Au. 2022 Apr 4;2(4):1007–17. doi: 10.1021/jacsau.2c00130 (PMC9088800; doi:10.1021/jacsau.2c00130)
Supplement: Supplementary file 1 — au2c00130_si_001.pdf [file au2c00130_si_001.pdf]

SUPPLEMENTARY INFORMATION

An expanded LUXendin color palette for GLP1R detection and visualization *in vitro* and *in vivo*

Julia Ast[1,2#], Alissa N. Novak[3,#], Tom Podewin[4], Nicholas H.F. Fine[1,2], Ben Jones[5], Alejandra Tomas[6], Ramona Birke[7], Kilian Roßmann[7], Bettina Mathes[4], Jenny Eichhorst[8], Martin Lehmann[8], Amelia K. Linnemann[3,*], David J. Hodson[1,2,9,*] and Johannes Broichhagen[4,7,*]

[1] Institute of Metabolism and Systems Research (IMSR), and Centre of Membrane Proteins and Receptors (COMPARE), University of Birmingham, Birmingham, B15 2TT, UK.
[2] Centre for Endocrinology, Diabetes and Metabolism, Birmingham Health Partners, Birmingham, B15 2TT, UK.
[3] Department of Pediatrics, and Indiana Center for Diabetes and Metabolic Diseases, Indiana University School of Medicine, Indianapolis, IN 46202, USA.
[4] Department of Chemical Biology, Max Planck Institute for Medical Research, 69120 Heidelberg, Germany.
[5] Imperial College London, Section of Endocrinology and Investigative Medicine, Division of Diabetes, Endocrinology and Metabolism, London, W12 0NN, UK.
[6] Section of Cell Biology and Functional Genomics, Division of Diabetes, Endocrinology and Metabolism, Imperial College London, London, W12 0NN, UK.
[7] Leibniz-Forschungsinstitut für Molekulare Pharmakologie, 13125 Berlin, Germany.
[8] Department of Pharmacology and Cell Biology, Leibniz-Forschungsinstitut für Molekulare Pharmakologie, 13125 Berlin, Germany.
[9] Oxford Centre for Diabetes, Endocrinology and Metabolism (OCDEM), NIHR Oxford Biomedical Research Centre, Churchill Hospital, Radcliffe Department of Medicine, University of Oxford, Oxford, OX3 7LE, UK.

[#]equal contributions

*Correspondence should be addressed to:
aklinnem@iu.edu, david.hodson@ocdem.ox.ac.uk, broichhagen@fmp-berlin.de

SUPPLEMENTARY METHODS

General chemistry

Solvents for chromatography and reactions were purchased HPLC grade (Sigma-Aldrich, 99.8%, extra dry over molecular sieves). Cy7-Maleimide (#25080) and Cy3-Maleimide (#21080) were purchased from Lumiprobe and CF488-Maleimide (#SCJ4600016-1UMOL) was purchased from Aldrich. All other reagents were used without further purification.

LC-MS was performed on an Agilent 1200 Infinity II LC System equipped with Thermo Scientific Accucore XL-C18 column (4 µm, 4.6 × 100 mm). Buffer A: 0.1% FA in H_2O Buffer B: 0.1% FA acetonitrile. The typical gradient was from 5% B for 0.5 min → gradient to 99% B over 5 min → 95% B for 0.5 min → gradient to 99% B over 1 min with 1.5 mL/min flow. Raw chromatograms were imported into and plotted in Graphpad Prism8. Injection peaks were also observed by running blank samples.

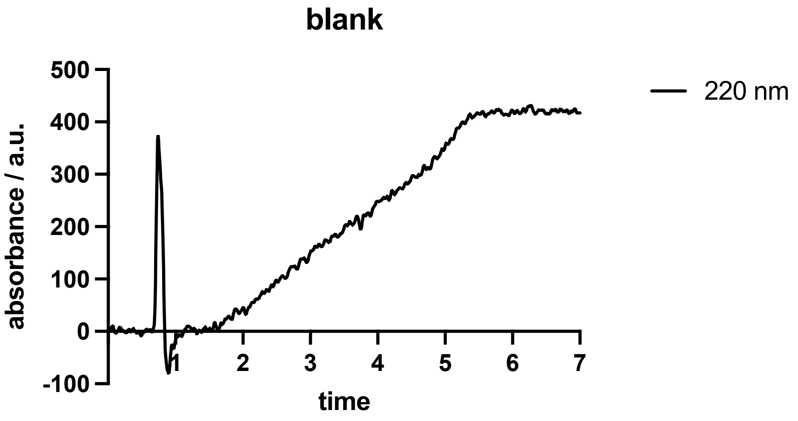

Preparative or semi-preparative HPLC was performed on an Agilent 1260 Infinity II LC System equipped with columns as followed: preparative column – Reprospher 100 C18 columns (10 µm: 50 x 30 mm at 20 mL/min flow rate; semi-preparative column – 5 µm: 250 x 10 mm at 4 mL/min flow rate. Eluents A (0.1% TFA in H_2O) and B (0.1% TFA in MeCN) were applied as a linear gradient. Peak detection was performed at maximal absorbance wavelength (at 220, 500, 550, 615 or 750 nm).

NMR spectra were recorded in deuterated solvents at 300 K at 750 MHz on Bruker AV-III spectrometers (Bruker Biospin, Rheinstetten, Germany) using cryogenically cooled 5 mm TCI-triple resonance probe equipped with one-axis self-shielded gradients and calibrated to residual solvent peaks (^{1}H in ppm): MeCN-d3 (1.94). The software used to control the spectrometer was topspin 3.5 pl6.Multiplicities are abbreviated as follows: s = singlet, d = doublet, t = triplet, q = quartet, br = broad, m = multiplet. Spectra are reported based on appearance, not on theoretical multiplicities derived from structural information.

High resolution mass spectrometry was performed on a Orbitrap Elite mass spectrometer (Thermo Fisher scientific) through direct infusion using a HESI source. The monoisotopic masses of the molecules were obtained by deconvoluting the mass spectrometry raw data using xtract algorithm that is built into FreeStyle software version 1.6 (Thermo Fisher scientific).

UV/Vis spectra were recorded on a Jasco V-770 UV/Vis/NIR Spectrophotometer (PbS-version) with a PAC-743 Peltierthermo 6/8 sample switching unit and a Julabo F-250 cooling system using Hellma quartz glass cuvettes (10 mm pathlength). Fluorescence excitation and emission spectra were recorded in Greiner black flat bottom 96 well plates on a TECAN INFINITE M PLEX plate reader. The spectra were recorded in PBS buffer pH 7.4.

Synthesis of CPY-Maleimide

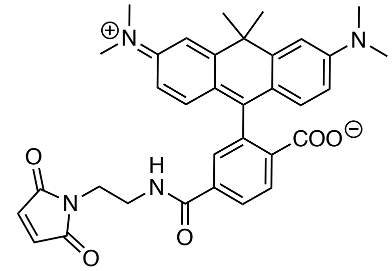

A 1 mL vial was charged with CPY-COOH (AAT Bioquest, custom synthesis) (135 µg, 296 nmol, 1.0 equiv.) in 100 µL DMF and DIPEA (2.59 µL, 1.18 µmol, 4.0 equiv.) was added before TSTU (98.0 µg, 326 nmol, 1.1 equiv.). After 15 min the mixture was added dropwise to a 1 mL vial charged with 1-(2-aminoethyl)pyrrole-2,5-dione hydrochloride (94.0 µg, 296 nmol, 1.0 equiv.) and DIPEA (2.59 µL, 1.18 µmol, 4.0 equiv.) in 100 µL DMF. After 30 min the reaction was quenched with 2% HOAc in H_2O:MeCN (50:50) and subjected to RP-HPLC to yield the desired product (108 µg, 236 nmol, 80%) as a blue powder after lyophilization.

^{1}H NMR (750 MHz, MeCN-d_3): δ [ppm] = 8.13 (d, J = 8.1 Hz, 1H), 7.92 (dd, J = 8.1, 1.6 Hz, 1H), 7.40 (s, 1H), 7.25 (d, J = 6.3 Hz, 1H), 7.06 (d, J = 2.6 Hz, 2H), 6.75 (d, J = 9.1 Hz, 2H), 6.69 (s, 1H), 6.66 (dd, J = 9.1, 2.6 Hz, 2H), 4.45 (s, 1H), 3.61 (t, J = 5.7 Hz, 2H), 3.45 (q, J = 5.9 Hz, 2H), 3.12 (s, 13H), 1.86 (s, 3H), 1.75 (s, 3H).

HRMS (ESI): calc. for $C_{34}H_{35}N_4O_5$ $[M+H]^+$: 579.2602, found: 579.2601.

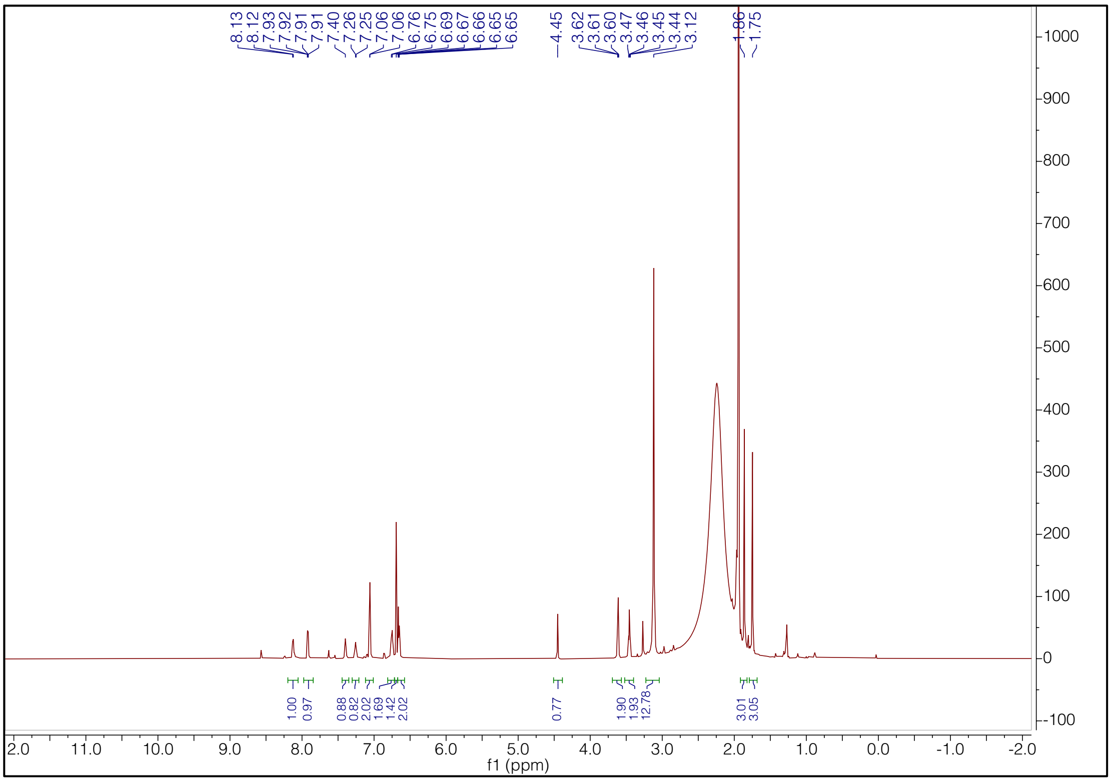

Synthesis of S39C-Ex4(9-39)

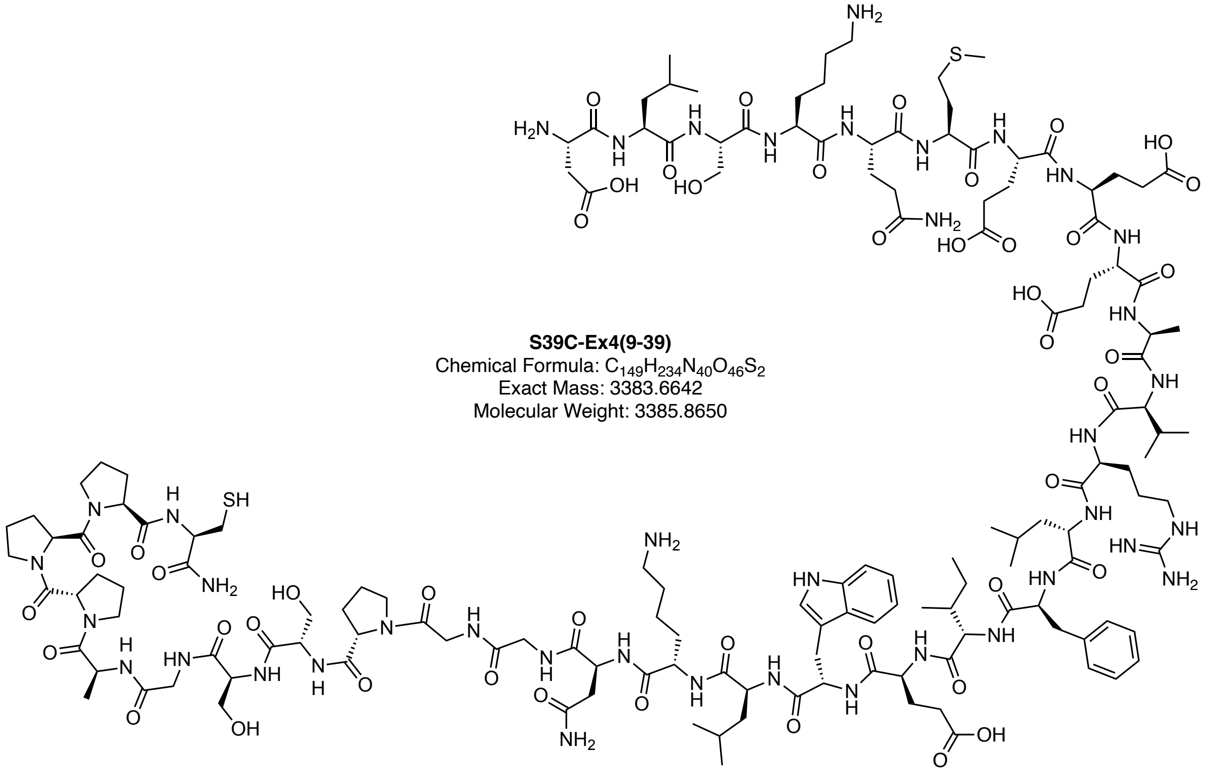

S39C-Ex4(9-39) peptide has been described previously.[1] Briefly, peptide was synthesized on a CEM Liberty Blue Peptide Synthesizer with a CEM Discovery Microwave using

standard Fmoc-protected solid phase peptide synthesis protocols with standard reagents. Pre-loaded Fmoc-Cys(Trt)-Tentagel S PHB resin (Rapp Polymere, Germany) containing 0.2-0.3 mmol/g amino acid was used as solid-phase. Peptide synthesis scale was 0.1 mmol using the standard coupling reagents DIC/Oxyma 0.5/1.0 M in DMF and DIPEA 2 M in DMF. Fmoc-protected amino acids (Sigma-Aldrich and NovaBiochem Merck, Germany) with standard residual protecting groups were coupled using a five-fold excess (2 M solutions). Deprotection of the Fmoc-protecting group was achieved by treatment with 20% piperidine in DMF. After completion of all coupling steps, the resin-bound peptide was transferred into a syringe with frit followed by global deprotection using 10 mL of a TFA:H_2O:tri-*iso*-propylsilane (95:2.5:2.5) mixture within 2 h under argon atmosphere. The peptide solution was filtered and the filtrate was concentrated under reduced pressure and residual TFA was removed by co-evaporation with toluene (3x). The residue was dissolved in a small amount of methanol and precipitated in 40 mL chilled diethyl ether and stored overnight at –38 °C to complete precipitation. The suspension was subjected to centrifugation (1843 x g, 4 °C, 5 min), the supernatant removed and the residue dried before being reconstituted in water and subjected to RP-HPLC purification. The combined purified fractions were lyophilized and the **S39C-Ex4(9-39)** peptide was obtained as a white TFA salt.

Synthesis of LUXendin492, LUXendin551, LUXendin615 and LUXendin762

To a solution of S39C-Ex4(9-39) (2.03 mg, 600 nmol, 1.0 eq.) in PBS (400 μL) was added 150 μL of a 10 mM TCEP x HCl solution in dH_2O and allowed to stand at r.t. for 5 minutes before the mixture was transferred to a fluorophore-maleimide (900 nmol, 1.5 eq.) containing solution in MeCN (400 μL). The reaction mixture was incubated at 38 °C overnight with gentle shaking before being subjected to RP-HPLC purification (water/ACN gradient, 90/10 → 10/90 in 60 min). The purified fractions were combined, the concentration was determined *via* the absorption of the fluorophore at the maximal wavelength and stocks of 10 nmol were prepared and lyophilized to yield **LUXendin492**, **LUXendin551**, **LUXendin615** and **LUXendin762** as their colored TFA salt. Storage was performed at -80 °C.

LUXendin492

The structure of the CF488A fluorophore remains undisclosed.

λ_{max} = 492 nm; ε = 70,000 cm^{-1} M^{-1}. Yield: 140 nmol; 23%.

HRMS (ESI): found: 4418.9749.

LUX492

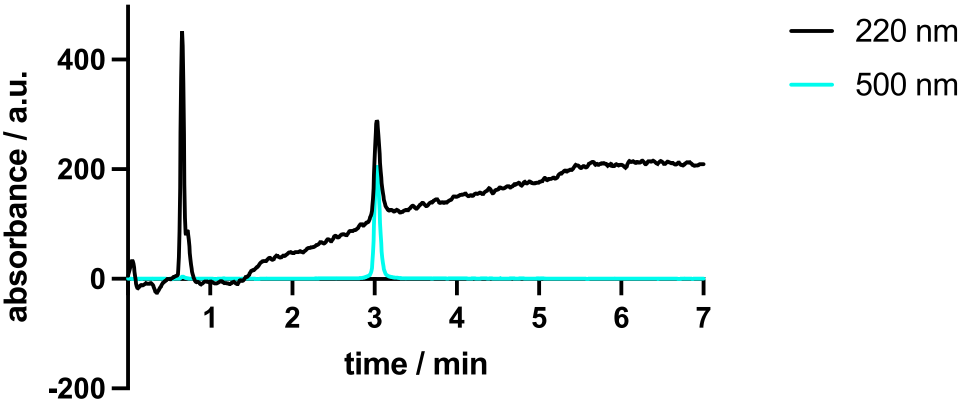

LUXendin551

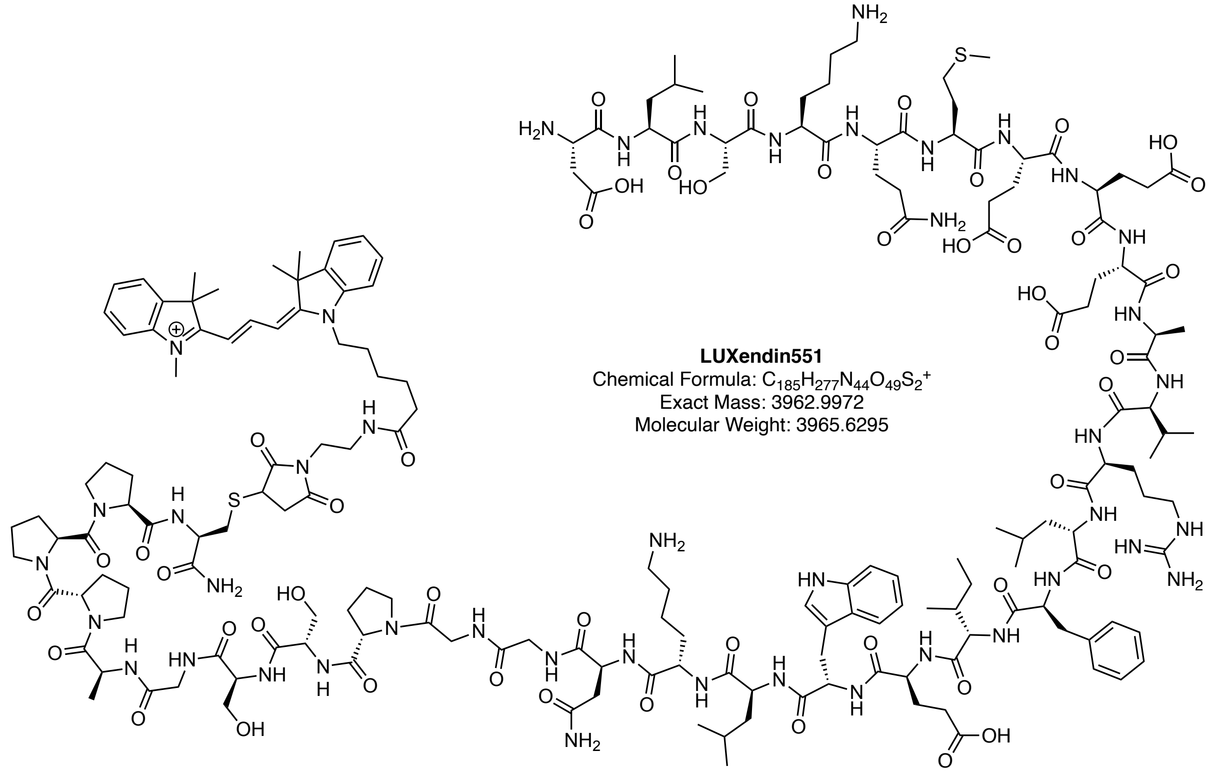

λ_{max} = 551 nm; ε = 150,000 cm^{-1} M^{-1}. Yield: 114 nmol; 19%.

HRMS (ESI): calc. for $C_{185}H_{277}N_{44}O_{49}S_2$ $[M]^+$: 3962.9972, found: 3962.9952.

LUX551

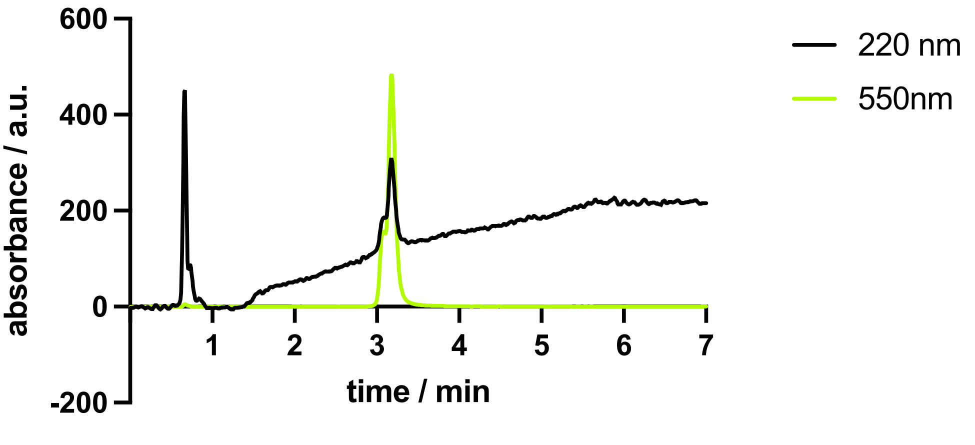

LUXendin615

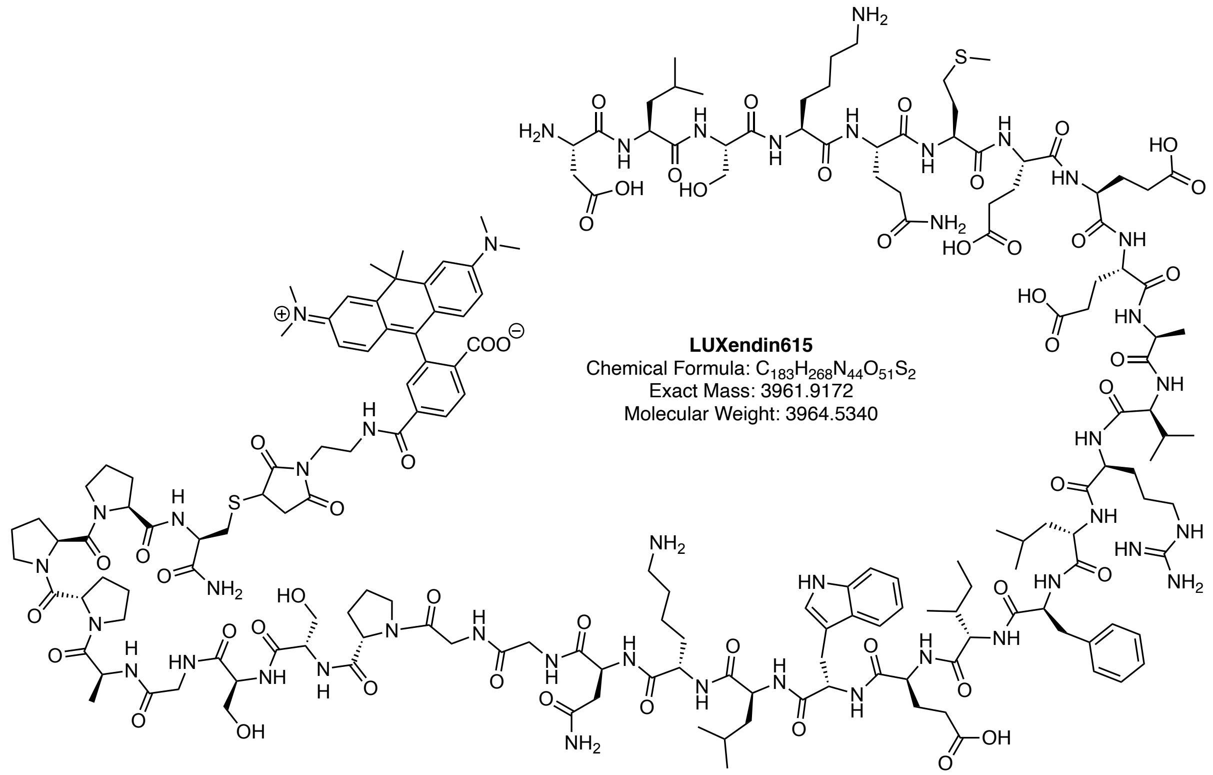

Reaction was performed with 150 nmol of peptide. Two peaks were detected via LCMS, which did separate more until longer incubation time, presumably accounting for different foldamers.

λ_{max} = 615 nm; ε = 100,000 cm^{-1} M^{-1}. Yield: 87 nmol; 58%.

HRMS (ESI): calc. for $C_{183}H_{268}N_{44}O_{51}S_2$ [M]: 3961.9172, found: 3961.9151.

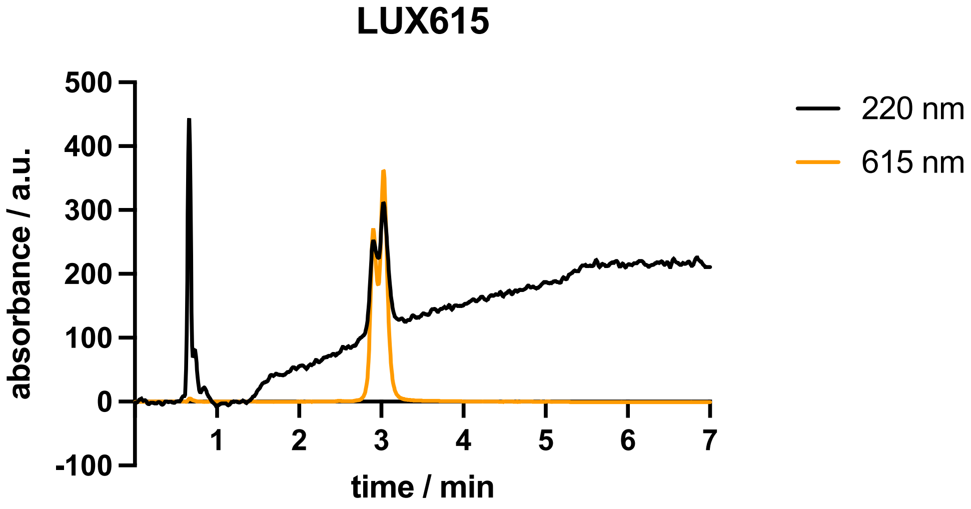

LUXendin762

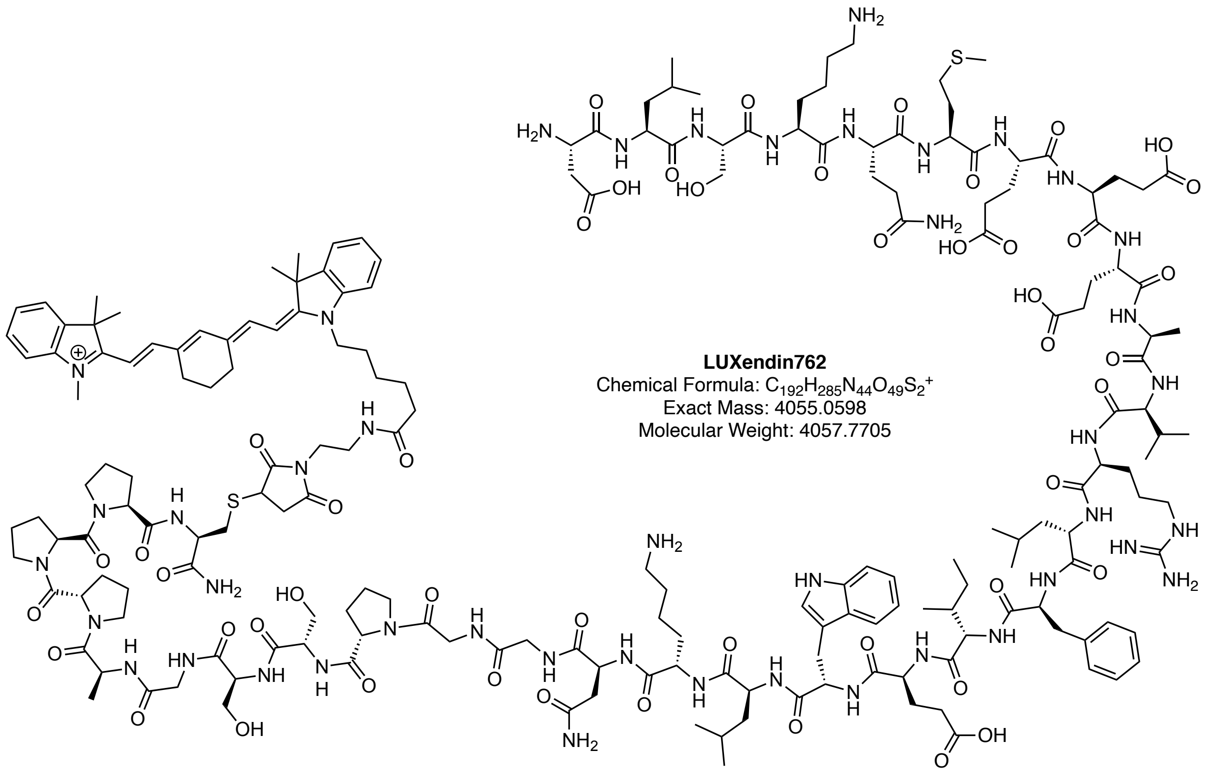

λ_{max} = 762 nm; ε = 199,000 cm^{-1} M^{-1}. Yield: 328 nmol; 55%.

HRMS (ESI): calc. for $C_{192}H_{285}N_{44}O_{49}S_2^+$ $[M]^+$: 4055.0598, found:4055.0530.

LUX762

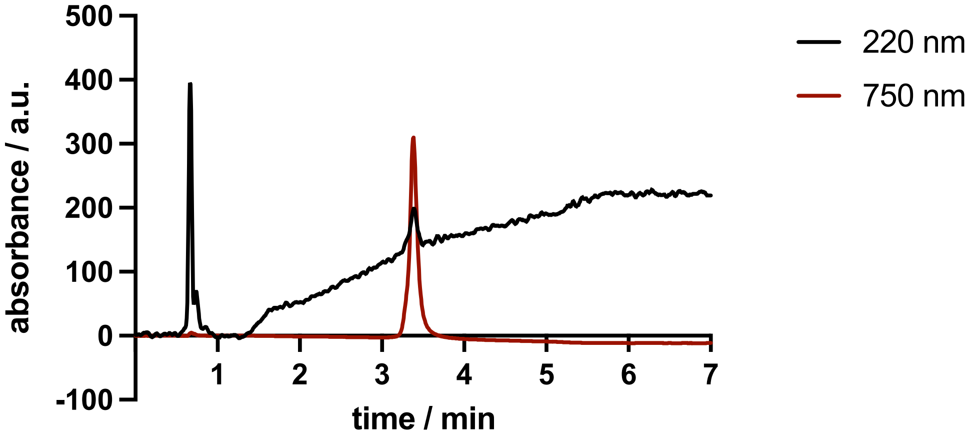

SUPPLEMENTARY FIGURES

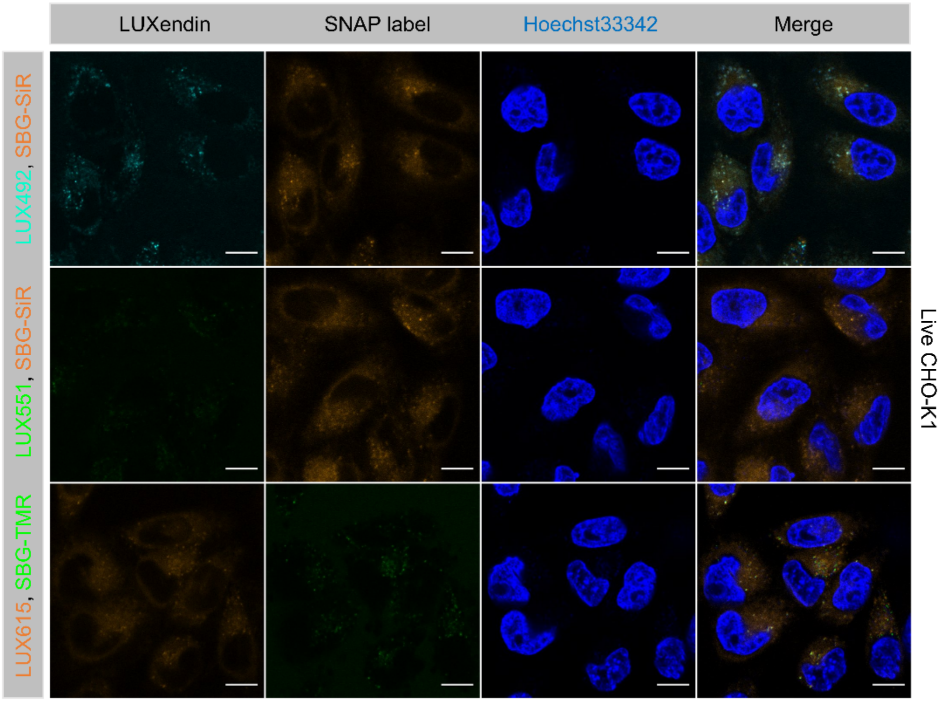

Supplementary Figure 1: Labeling of live CHO-K1 cells with LUXendin492, LUXendin551 and LUXendin615. CHO-K1 cells were treated with **LUXendin492** (LUX492), **LUXendin551** (LUX551) and **LUXendin615** (LUX615), the same way and at the same time as SNAP-GLP1R:CHO-K1 in Figure 2 (scale bar = 10 µm).

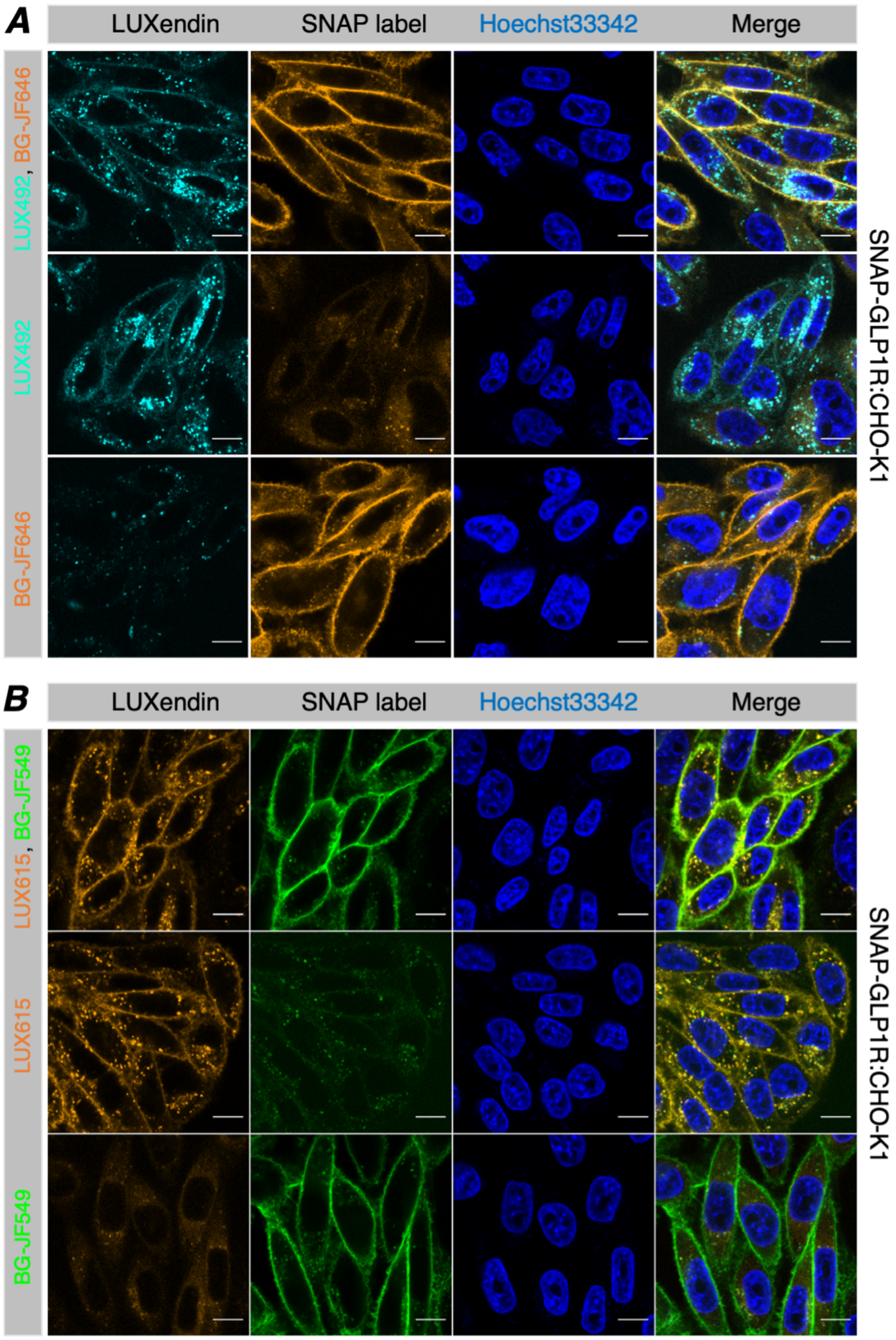

Supplementary Figure 2: Labeling of live cells with LUXendin492 and LUXendin615 and cell permeable SNAP label. SNAP-GLP1R:CHO-K1 cells were incubated with **LUXendin492** (LUX492) in addition to orthogonal SNAP-labeling with cell-permeable BG-JF_{646} and confocal imaging (nuclei were stained using Hoechst33342) (scale bar = 10 µm). **B)** SNAP-GLP1R:CHO-K1 cells were incubated with **LUXendin615** (LUX615) in addition to orthogonal SNAP-labeling with cell-permeable BG-JF_{549} and confocal imaging (nuclei were stained using Hoechst33342) (scale bar = 10 µm).

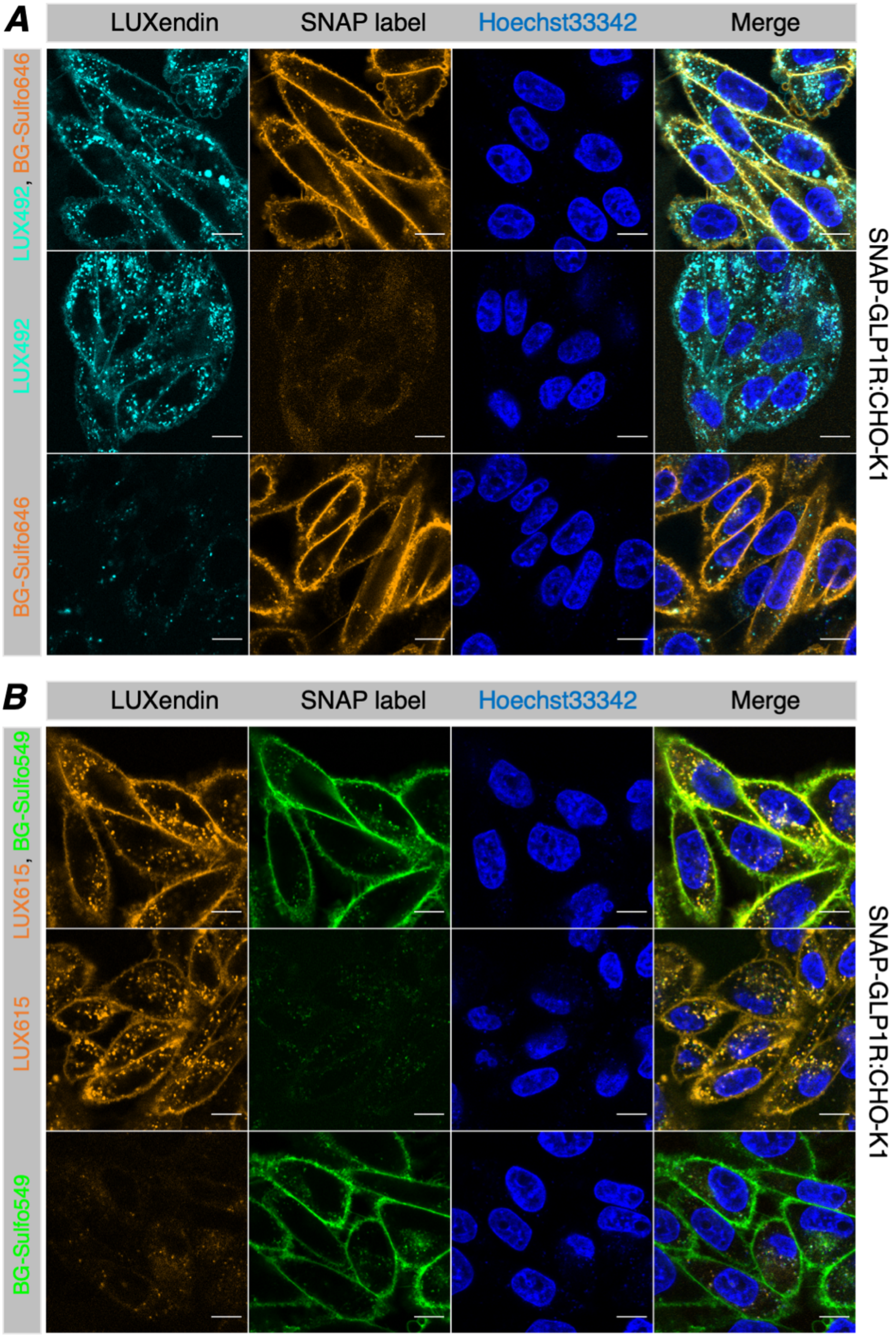

Supplementary Figure 3: Labeling of live cells with cell impermeable SNAP label chased by LUXendin492 and LUXendin615. SNAP-GLP1R:CHO-K1 cells were incubated with **LUXendin492** (LUX492) in addition to orthogonal SNAP-labeling with cell-impermeable BG-Sulfo646 and confocal imaging (nuclei were stained using Hoechst33342) (scale bar = 10 µm). **B)** SNAP-GLP1R:CHO-K1 cells were incubated with **LUXendin615** (LUX615) in addition to orthogonal SNAP-labeling with cell-impermeable BG-Sulfo549 and confocal imaging (nuclei were stained using Hoechst33342) (scale bar = 10 µm).

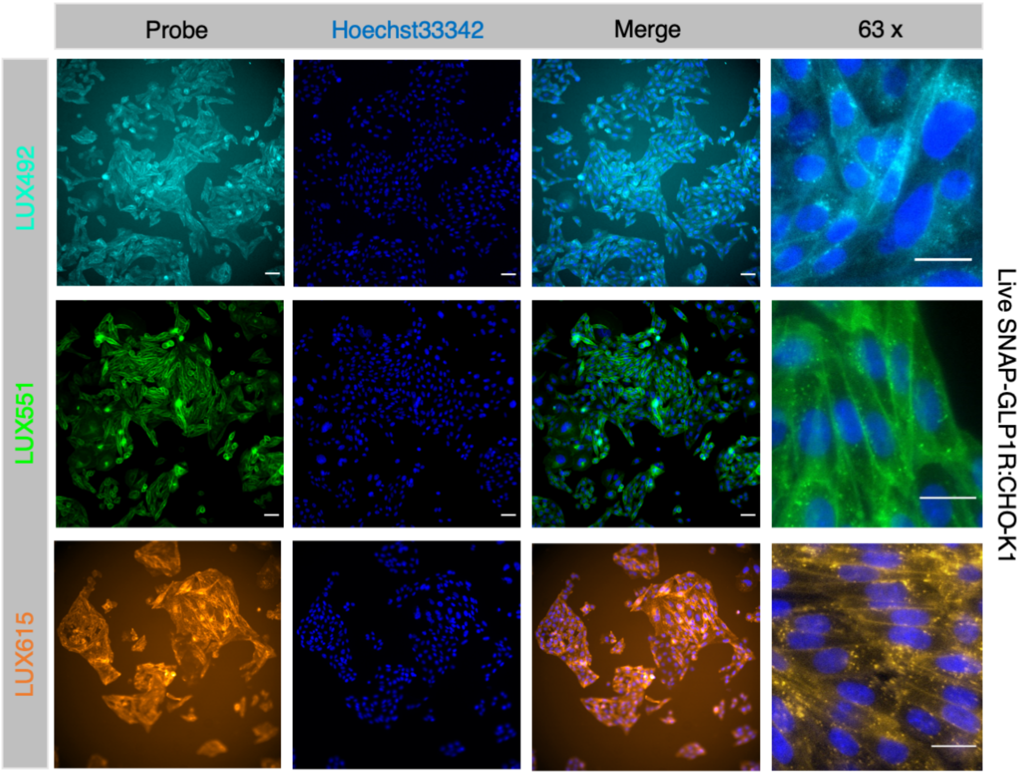

Supplementary Figure 4: **Widefield imaging of LUXendin492, LUXendin551 and LUXendin615 labeling.** SNAP-GLP1R:CHO-K1 were labeled with **LUXendin492** (LUX492), **LUXendin551** (LUX551) and **LUXendin615** (LUX615), before widefield imaging (scale bar = 50 µm) (n = 3 independent experiments). Nuclei were stained using Hoechst33342.

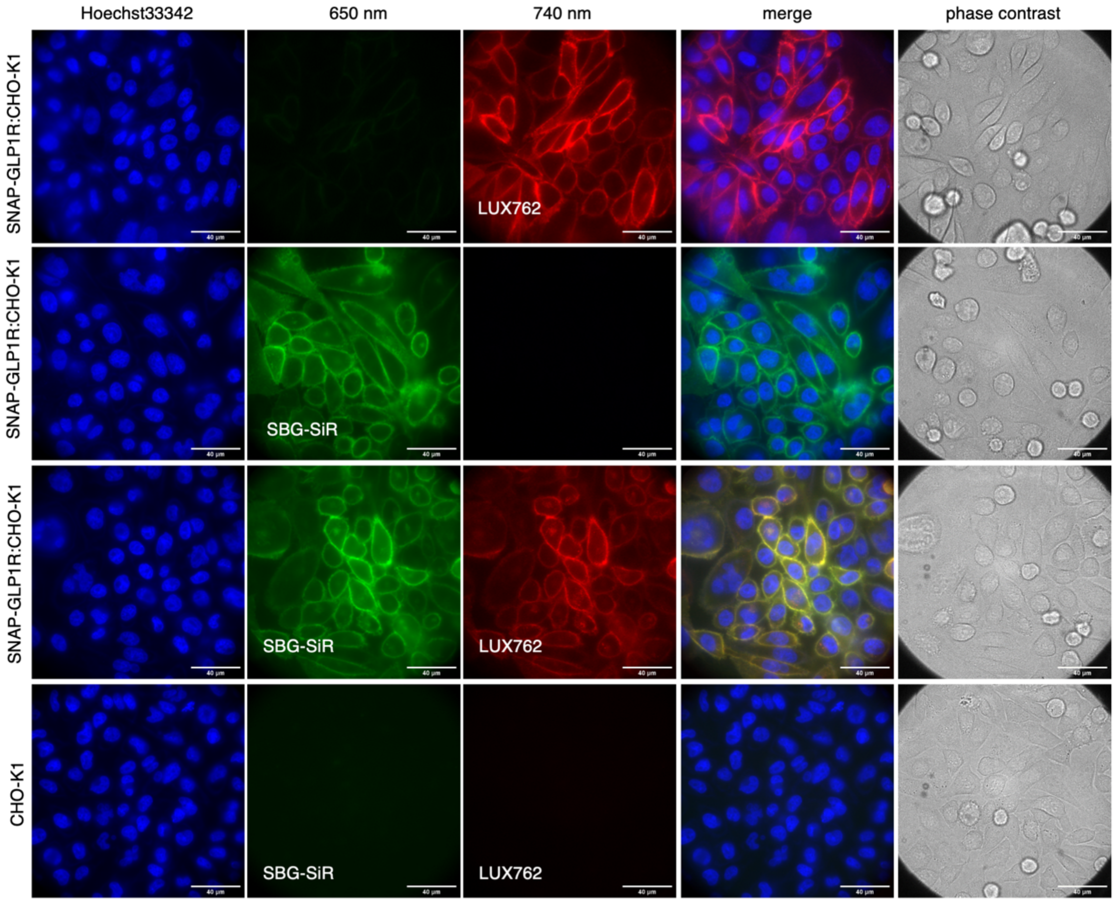

Supplementary Figure 5: Widefield imaging of LUXendin762 labeling. SNAP-GLP1R:CHO-K1 were labeled with **LUXendin762** and SBG-SiR before widefield imaging (scale bar = 40 µm) (n = 3 independent experiments). Nuclei were stained using Hoechst33342 and CHO-K1 cells were used as controls.

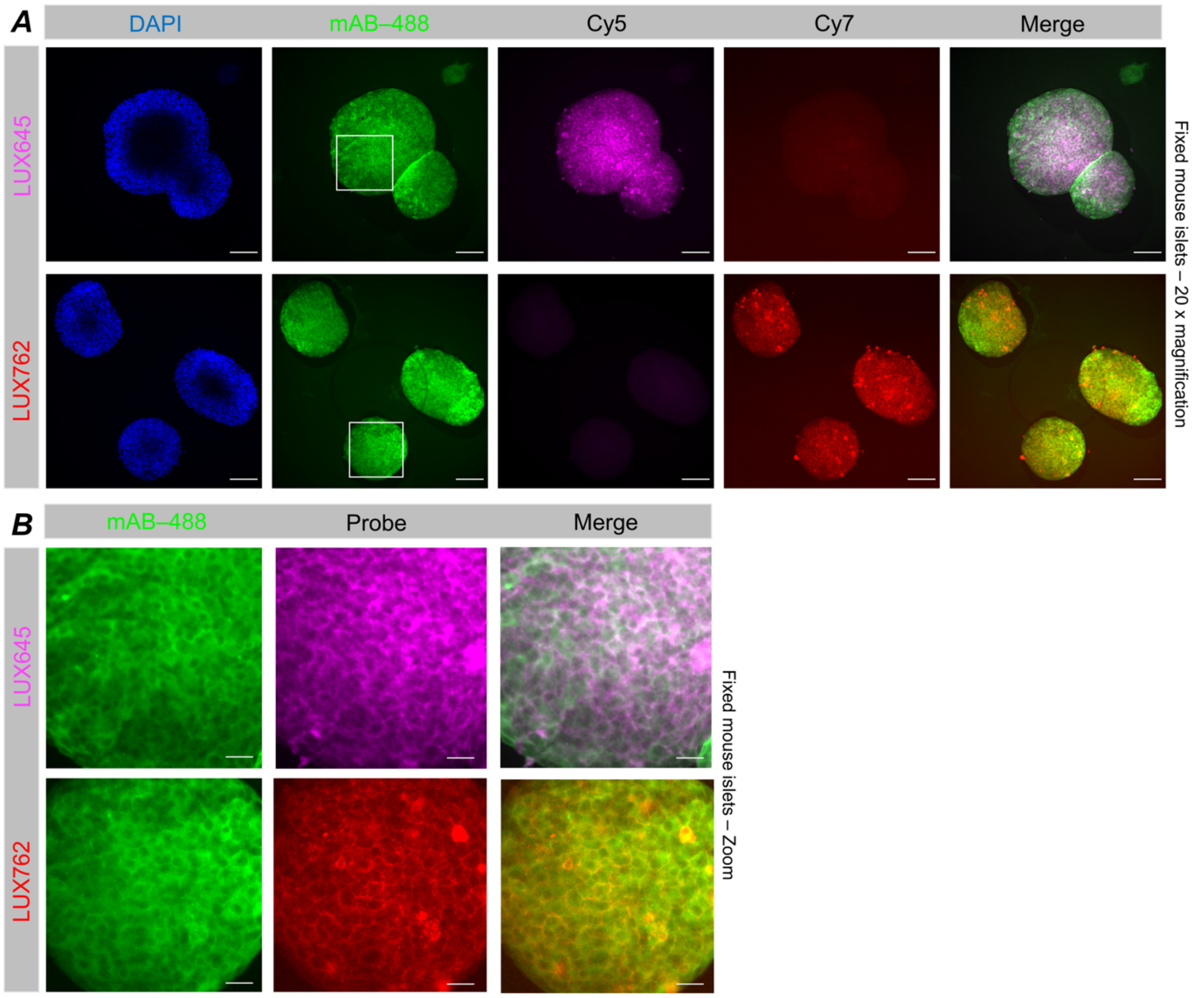

Supplementary Figure 6: Labeling of fixed islets of Langerhans with LUXendin762. A) Islets labeled with **LUXendin762** (LUX762) can be formaldehyde fixed, allowing co-staining with GLP1R monoclonal antibody (mAb-488). **LUXendin645** (LUX645) was used as a known positive control (scale bar = 100 µm) (n > 5 islets from 3 animals). **B)** Zoom-in from **A**) showing overlap between **LUXendin762**/mAb-488 and **LUXendin645**/mAb-488 (scale bar = 25 µm) (n > 5 islets from four animals).

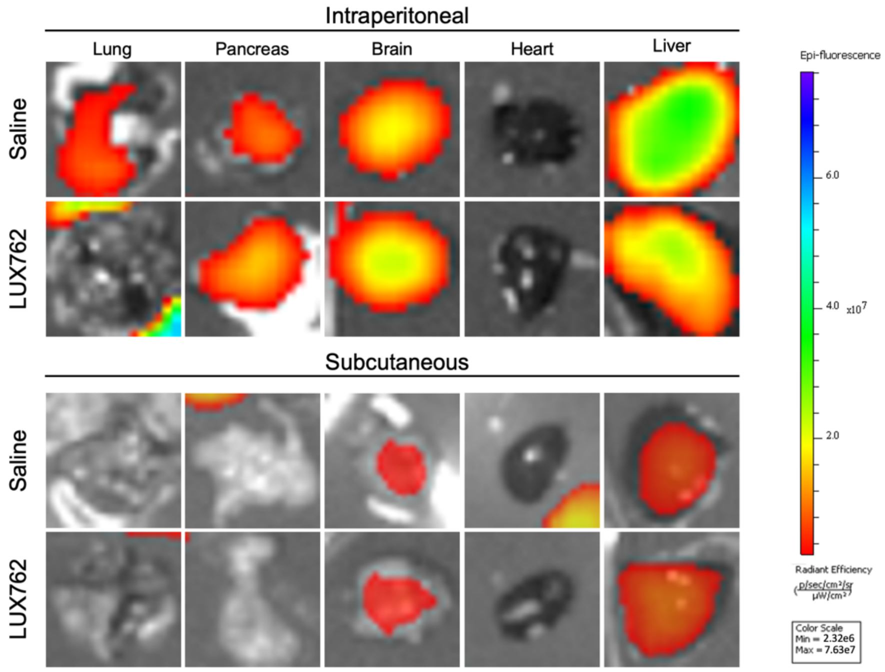

Supplementary Figure 7: Images of individual tissue treated with LUXendin762.

REFERENCES

[1] J. Ast, A. Arvaniti, N. H. F. Fine, D. Nasteska, F. B. Ashford, Z. Stamataki, Z. Koszegi, A. Bacon, B. J. Jones, M. A. Lucey, S. Sasaki, D. I. Brierley, B. Hastoy, A. Tomas, G. D'Agostino, F. Reimann, F. C. Lynn, C. A. Reissaus, A. K. Linnemann, E. D'Este, D. Calebiro, S. Trapp, K. Johnsson, T. Podewin, J. Broichhagen, D. J. Hodson, *Nat Commun* **2020**, *11*, 467.
